# LINC02167 stabilizes KSR1 mRNA in an m^5^C-dependent manner to regulate the ERK/MAPK signaling pathway and promotes colorectal cancer metastasis

**DOI:** 10.1186/s13046-025-03368-w

**Published:** 2025-04-15

**Authors:** Junwen Qi, Tao Jiang, Bowen Liu, Qihang Hu, Junnan Chen, Ning Ma, Yixin Xu, Hu Song, Jun Song

**Affiliations:** 1 Department of General Surgery, The Affiliated Hospital of Xuzhou Medical University, Xuzhou, Jiangsu, 221006 China; 2 Institute of Digestive Diseases, Xuzhou Medical University, Xuzhou, Jiangsu, 221002 China; 3 Affiliated First Clinical College, Xuzhou Medical University, Xuzhou, Jiangsu, 221004 China; 4 Central Laboratory, The Affiliated Hospital of Xuzhou Medical University, Xuzhou, Jiangsu, 221002 China

**Keywords:** Long noncoding RNA, Metastasis, KSR1, 5-methylcytosine, Transcription

## Abstract

**Background:**

Metastasis is a leading cause of colorectal cancer (CRC)-related mortality, yet its molecular mechanisms remain poorly understood. Long noncoding RNAs (lncRNAs) have emerged as critical regulators of CRC metastasis, but their specific roles are not fully elucidated. This study identifies and characterizes a novel lncRNA LINC02167 as a critical regulator of CRC metastasis.

**Methods:**

LINC02167 expression was analyzed in CRC tissues via real-time quantitative polymerase chain reaction and fluorescence in situ hybridization. Functional assays evaluated its role in CRC cell migration, invasion, and metastasis in vitro and in vivo. Mechanistic exploration involves a combination of techniques, including RNA sequencing, mass spectrometry, RNA pull-down, RNA immunoprecipitation, chromatin immunoprecipitation, luciferase reporter assays, RNA stability assays, and bioinformatics analysis, to uncover the molecular interactions and pathways regulated by LINC02167.

**Results:**

LINC02167 is markedly upregulated in CRC tissues and strongly correlates with advanced clinical features and poor prognosis. Functional analyses reveal that LINC02167 enhances CRC cell migration and invasion in vitro and promotes metastasis in vivo. Mechanistically, LINC02167 serves as a molecular scaffold, forming a complex with YBX1 and ILF3 to facilitate YBX1 binding to NSUN2-mediated m^5^C modification sites on KSR1 mRNA, thereby stabilizing KSR1 mRNA and activating the ERK/MAPK signaling pathway to drive CRC metastasis. Additionally, MYC-driven transcriptional activation leads to the upregulation of LINC02167 in CRC.

**Conclusions:**

This study uncovers a novel mechanism through which LINC02167 promotes the ERK/MAPK pathway and CRC metastasis via m^5^C modification, underscoring its potential as a promising therapeutic target for metastatic CRC treatment.

**Supplementary Information:**

The online version contains supplementary material available at 10.1186/s13046-025-03368-w.

## Background

Colorectal cancer (CRC) is the third most commonly diagnosed malignancy worldwide and the second leading cause of cancer-related mortality [1, 2]. At diagnosis, 20–25% of CRC patients present with distant metastases, and nearly half will develop metastatic disease during disease progression [3]. The five-year survival rate for metastatic CRC is below 15%, compared to over 90% for localized disease [4]. This dramatic disparity in survival outcomes underscores the urgent need to elucidate metastatic mechanisms and identify novel therapeutic targets to improve prognosis for advanced CRC patients.

Long non-coding RNAs (lncRNAs) have emerged as pivotal regulators in cancer biology, driving tumor metastasis through epigenetic modifications as well as transcriptional and post-transcriptional regulation [5]. In CRC, lncRNAs often promote metastasis by interacting with RNA-binding proteins, thereby modulating downstream gene expression and signaling pathways. For example, lncRNA SNHG1 binds to the HNRNPD protein to stabilize SERPINA3 mRNA, facilitating CRC metastasis [6]. LOC101928222 promotes tumor progression by stabilizing HMGCS2 mRNA and enhancing cholesterol biosynthesis [7]. These findings underscore the need to further explore the distinct roles and regulatory mechanisms of lncRNAs in CRC metastasis.

As a critical component of the epigenetic regulatory network, 5-methylcytosine (m^5^C) is implicated in various cellular processes and systemic diseases, including cell migration and cancer metastasis [8, 9]. This modification is catalyzed by RNA methyltransferases such as NSUN2, which add methyl groups to cytosine residues, and is dynamically regulated by demethylases (e.g., the TET family) and binding proteins (e.g., ALYREF and YBX1) [10, 11]. Recent studies have shown that m^5^C modifications can promote cancer progression by enhancing the stability of mRNAs of genes such as ENO1 [12], HIF1A [13], and SKIL [14] in CRC. Additionally, lncRNAs have been shown to influence the degradation of methyltransferases like NSUN2, thereby affecting m^5^C modification of target mRNAs and facilitating cancer metastasis [15]. Despite these findings, the regulatory roles and mechanisms of m^5^C in CRC, particularly its interplay with lncRNAs, remain incompletely understood and warrant further investigation.

The ERK/MAPK signaling pathway is pivotal in CRC progression and metastasis, regulating critical processes such as cell proliferation, survival, and migration [16]. Approximately 50% of CRC patients harbor mutations in genes associated with this pathway [17]. Consequently, targeting ERK/MAPK signaling has become a central focus of cancer research [18–20]. While core components of the pathway, including RAF, MEK, and ERK, have been directly targeted in therapeutic strategies, these treatments face challenges such as systemic toxicity, pathway reactivation, or activation of compensatory pathways [21, 22]. Kinase Suppressor of Ras 1 (KSR1), a scaffold protein within this pathway, is essential for maintaining the RAF-MEK-ERK cascade, ensuring efficient and specific signal transduction [23, 24]. Despite its established role, the functions of KSR1 in CRC metastasis remain poorly understood. Further investigation into KSR1 and its upstream regulatory mechanisms may provide novel approaches to modulate ERK/MAPK signaling and overcome the limitations of direct kinase inhibitors.

This study investigates the role of the novel lncRNA LINC02167 in CRC metastasis, focusing on its mechanism of stabilizing KSR1 mRNA in an m^5^C manner. Additionally, we examine the MYC-driven transcriptional regulation of LINC02167. These findings aim to provide insights into the mechanistic functions of lncRNAs and m^5^C modifications in CRC, highlighting their potential as therapeutic targets for metastatic disease.

## Materials and methods

### Clinical specimens

This study was approved by the Ethics Committee of the Affiliated Hospital of Xuzhou Medical University (approval number: XYFY2020-KL185-01), and informed consent was obtained from all participants. A cohort of 80 matched pairs of CRC tissues and adjacent normal tissues (ANTs) was collected from patients undergoing radical or palliative resection in the Department of Gastrointestinal Surgery, Affiliated Hospital of Xuzhou Medical University. These samples were used for RNA extraction to assess LINC02167 expression levels. Plasma samples from 28 non-metastatic CRC patients and 16 metastatic CRC patients were collected to measure LINC02167 levels. Additionally, a separate cohort of 116 matched paraffin-embedded CRC and ANT samples was acquired for tissue microarray (TMA) analysis. All patients had a pathologically confirmed CRC diagnosis and did not receive preoperative radiotherapy or chemotherapy. Clinicopathological data, including patient age, sex, tumor size, and depth of invasion, were collected.

### Transwell migration and invasion assays

Transwell assays were conducted using 8-μm pore size chambers (Corning, Cat# 3422) following the manufacturer's protocol. For invasion assays, the upper chamber was pre-coated with Matrigel (MCE, Cat# HY-K6001), whereas for migration assays, no coating was applied. After overnight serum starvation, transfected CRC cells were seeded into the upper chamber, with complete medium added to the lower chamber. Cells were incubated at 37 °C with 5% CO₂ for 24–48 h. Migrated and invaded cells were then fixed, stained, and quantified.

### Wound healing assays

For wound healing assays, transfected CRC cells were seeded in six-well plates and cultured to approximately 90% confluency. A 10-μL pipette tip was used to create a linear scratch in each well, and exfoliated cells were removed by washing twice with PBS. Cells were then cultured in FBS-free medium. Migration distance was measured by capturing images at 0 h and 24 h using an inverted light microscope (Olympus, Tokyo, Japan).

### Biotinylated RNA pull-down assay

In brief, T7 promoter-containing DNA was amplified by PCR and purified using the TIANgel Purification Kit (TIANGEN BIOTECH, Cat# DP219-02). The purified DNA was incubated with a biotin RNA labeling mixture (Roche, Cat# 11,685,597,910), T7 RNA polymerase (Thermo Fisher Scientific, Cat# EP0111), and an RNase inhibitor at 37 °C for 2 h in a PCR thermal cycler. Biotin-labeled RNA probes were then purified using the RNAclean Kit (TIANGEN BIOTECH, Cat# DP412). Biotin-labeled RNA probes were incubated with protein extracts from CRC cells and subsequently mixed with precleared streptavidin agarose resin (Thermo Fisher Scientific, Cat# 20,347). RNA–protein complexes were isolated by centrifugation, and bound proteins were eluted, denatured, and subjected to Western blot analysis to detect the target proteins.

### Luciferase reporter assay

To explore the transcriptional regulation of LINC02167, two fragments of its promoter region were PCR-amplified and cloned into the pGL3-Basic luciferase vector. Cells with MYC knockdown or overexpression were transfected with these constructs. After 48 h, firefly luciferase activity was quantified using a Dual Luciferase Reporter Assay Kit (Beyotime, Cat# RG027), with Renilla luciferase as a normalization control.

#### Chromatin immunoprecipitation (ChIP) assay

ChIP assays were conducted using the BeyoChIP™ ChIP Assay Kit (Beyotime, Cat# P2078) following the manufacturer's protocol. Briefly, 1 × 10⁷ CRC cells were cross-linked with 1% formaldehyde for 15 min at room temperature, quenched with 0.125 M glycine, and lysed to isolate chromatin. DNA was fragmented to 200–1000 bp by sonication. Cross-linked protein-DNA complexes were immunoprecipitated with anti-MYC (Proteintech, Cat# 10,828–1-AP, RRID: AB_2148585) antibody or control IgG (Cell Signaling Technology, Cat# 2729, RRID: AB_1031062) at 4 °C overnight, followed by Protein A/G Magnetic Bead (MCE, Cat# HY-K0202) incubation for 2 h. After reversing cross-links with 5 M NaCl, DNA was purified (PCR Clean-Up Kit, Beyotime, Cat# D0033) and analyzed by qRT-PCR using primers P1–P9 (Additional file 1: Table S3).

### RNA stability assay

For the RNA stability assay, Actinomycin D (MCE, Cat# HY-17559) and 5,6-Dichlorobenzimidazole riboside (DRB) (MCE, Cat# HY-14392) were used to inhibit RNA synthesis. Briefly, transfected CRC cells were treated with 2 μg/ml of Actinomycin D or 25 μM of DRB to terminate transcription. Cells were then harvested at 0 h, 2 h, 4 h, 8 h, and 12 h. Subsequently, RNA was extracted from the cells and analyzed by qRT-PCR.

### Dot blot assay

Total RNA was collected and extracted, and a specified amount of RNA was spotted onto a nylon membrane followed by UV crosslinking for 30 min. The membrane was stained with 0.1% methylene blue (MB) as a loading control. Subsequently, the membrane was blocked with 5% skimmed milk for 1 h and incubated with an m^5^C antibody (1:1,000, Abcam, Cat# ab10805, RRID: AB_442823) overnight at 4℃. After washing with TBST buffer, the membrane was incubated with a horseradish peroxidase-conjugated secondary antibody at room temperature for 1 h. Signal detection was performed using an enhanced ECL chemiluminescent substrate kit (Vazyme, Cat# E411-05).

### Animal experiments

Female BALB/c nude mice ranging from 4 to 6 weeks old were obtained from Beijing Vital River Laboratory Animal Technology Co., Ltd. (Beijing, China), and housed under specific pathogen-free conditions. HCT116 cells stably transfected with LINC02167-targeting shRNA (Luc-sh-LINC02167) or non-specific control (Luc-sh-Ctrl) were validated and prepared. Mice were anesthetized with 1% sodium pentobarbital via intraperitoneal injection, and a left oblique incision was made to expose the spleen. HCT116 cells (1 × 10⁶) were slowly injected into the distal spleen. Following injection, the injection site was compressed with a 75% ethanol-soaked cotton swab for 2 min to prevent cell leakage. The spleen was then repositioned, and the incision was sutured. 60 days post-injection, liver metastasis of CRC cells was visualized using the Xenogen IVIS Spectrum imaging system (PerkinElmer, USA). The Animal Care and Use Committee at Xuzhou Medical University provided the ethics approval statements for all the animal experiments in this study.

### Statistical analysis

Statistical analyses were performed using SPSS 19.0 software (IBM, Armonk, NY, USA) and GraphPad Prism version 8.0 (La Jolla, CA, USA). All the data in the present study is presented as the means ± standard deviations (SD), and all the tests were two-sided. A *P* value < 0.05 was considered wo indicate statistical significance. Student’s t-test or one-way analysis of variance (ANOVA) was used to evaluate the significance of differences between groups. Correlations were detected by Spearman’s correlation coefficient. Overall survival (OS) and disease-free survival (DFS) were assessed by the Kaplan–Meier method and log-rank test. The relationship between LINC02167 expression and the clinicopathological parameters of CRC patients was calculated by the chi-square test or Fisher’s exact test. Univariate and multivariate Cox proportional hazard regression models were used to evaluate the effects of LINC02167 expression or other clinicopathological parameters on survival and the hazard ratio (HR).

### Additional methods

Additional experiments, including cell lines and culture, cell transfection, RNA extraction, qRT–PCR, nuclear–cytoplasmic separation, immunohistochemistry (IHC), RNA stability assays, fluorescence in situ hybridization (FISH), immunofluorescence (IF) staining, Western blot, methylated RNA Immunoprecipitation (MeRIP)-qPCR, RNA immunoprecipitation (RIP)-qPCR, Co-immunoprecipitation (Co-IP) assay, RNA sequencing, comprehensive identification of RNA-Binding proteins by mass spectrometry (ChIRP-MS), molecular docking and bioinformatics analysis are described in Additional file 2: Supplemental Materials and Methods.

## Results

### LINC02167 is upregulated in CRC and associated with advanced clinical features and poor prognosis

**Fig. 1:**
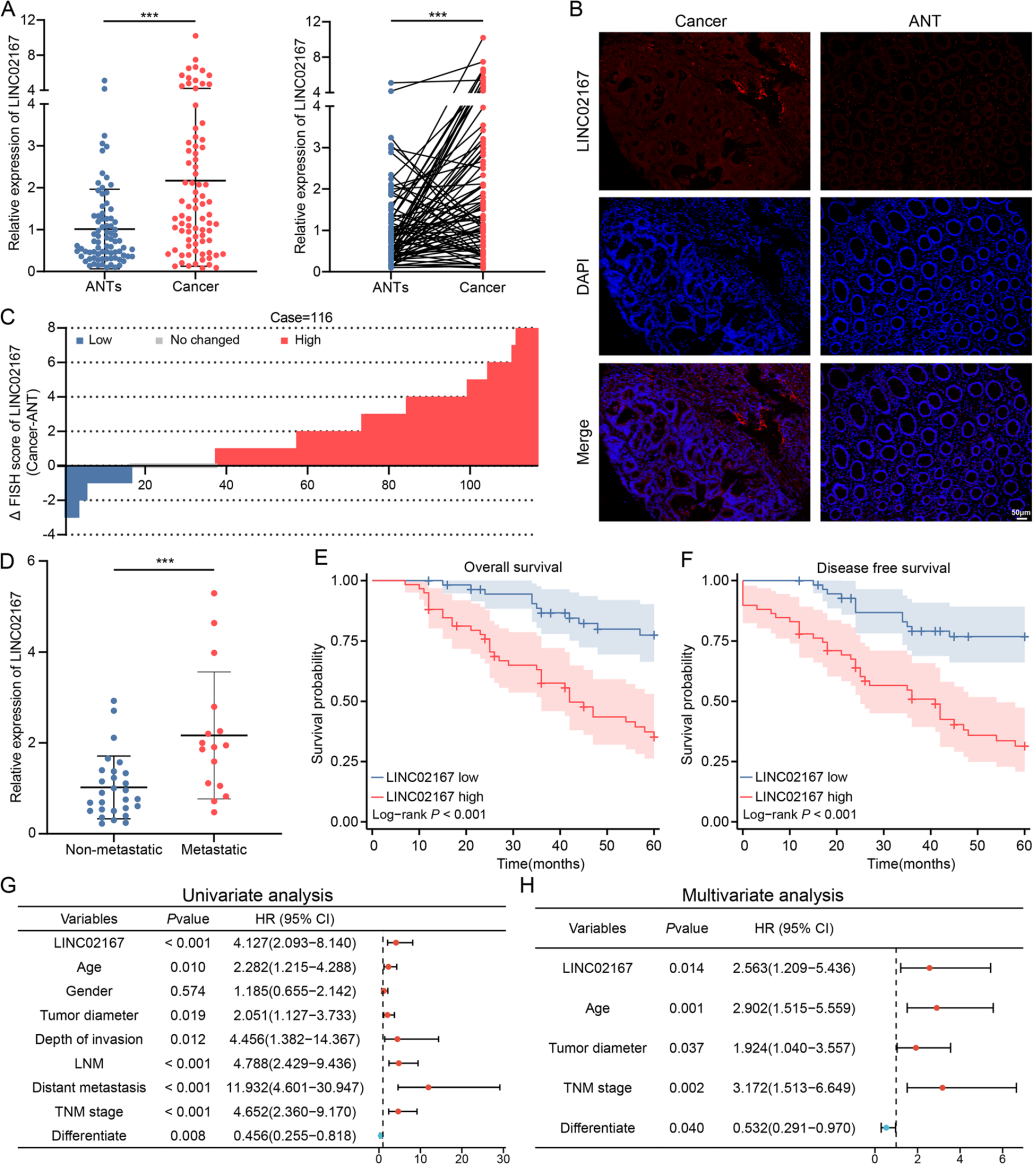
LINC02167 is upregulated in CRC and associated with advanced clinical features and poor prognosis. **A** qRT-PCR analysis of LINC02167 expression levels in CRC tissues and paired ANTs from clinical samples (*n* = 80). **B** Representative FISH images showing LINC02167 expression in CRC tissues and paired ANT from the tissue microarray (TMA) cohort. **C** Differences in FISH staining scores for LINC02167 between CRC tissues and paired normal tissues in the TMA cohort (*n* = 116). **D** qRT-PCR analysis of LINC02167 expression levels in plasma from metastatic CRC patients (*n* = 16) and non-metastatic CRC patients (*n* = 28). **E** Kaplan–Meier survival analysis showing OS in CRC patients from the TMA cohort based on LINC02167 expression levels. **F** Kaplan–Meier analysis of DFS in CRC patients from the TMA cohort stratified by LINC02167 expression levels. **G**, **H** Univariate and multivariate Cox regression analysis of LINC02167 expression and clinicopathological variables for OS prediction in CRC patients from the TMA cohort. All bar graphs represent 95% confidence intervals (CIs). HR, hazard ratio; CI, confidence interval. *P < 0.05, **P < 0.01, ***P < 0.001

Given that LINC02167 is a novel, insufficiently characterized lncRNA, was first analyzed for its expression in CRC tissues and paired ANTs using qRT-PCR. The results demonstrated a significant overexpression of LINC02167 in CRC tissues (Fig. 1A). Consistently, FISH analysis with a LINC02167-specific probe on TMA from 116 pairs of CRC tissues and paired ANTs confirmed its upregulation in CRC tissues (Fig. 1B-C). To investigate the clinical relevance of LINC02167 in CRC progression and diagnosis, the TMA patient cohort was stratified into high-expression and low-expression groups based on FISH staining scores, followed by clinical correlation analysis. Chi-square tests revealed significant associations between high LINC02167 expression and increased tumor invasion depth (*P* = 0.029), lymph node metastasis (*P* < 0.001), distant metastasis (*P* = 0.040), and advanced TNM stage (*P* < 0.001) (Additional file 1: Table S1). Additionally, plasma samples from metastatic and non-metastatic CRC patients were analyzed using qRT-PCR. LINC02167 levels were significantly elevated in the plasma of metastatic CRC patients compared to non-metastatic patients (Fig. 1D). These results suggest that high LINC02167 expression is closely associated with advanced metastasis in CRC patients.

The Kaplan–Meier survival analysis revealed that CRC patients with elevated LINC02167 expression in the TMA cohort experienced significantly shorter OS (Fig. 1E) and DFS (Fig. 1F). Furthermore, univariate Cox regression analysis suggested that LINC02167 expression, age, tumor diameter, depth of invasion, lymph node metastasis, distant metastasis, TNM stage, and differentiate were significant risk factors in terms of OS (Fig. 1G), while multivariate Cox analysis showed that LINC02167 expression, age, tumor diameter, TNM stage, and differentiate could serve as independent prognostic factors in terms of OS in CRC patients (Fig. 1H). These results highlight the pivotal role of LINC02167 as a prognostic biomarker for CRC.

### LINC02167 promotes CRC metastasis both in vitro and in vivo

**Fig. 2:**
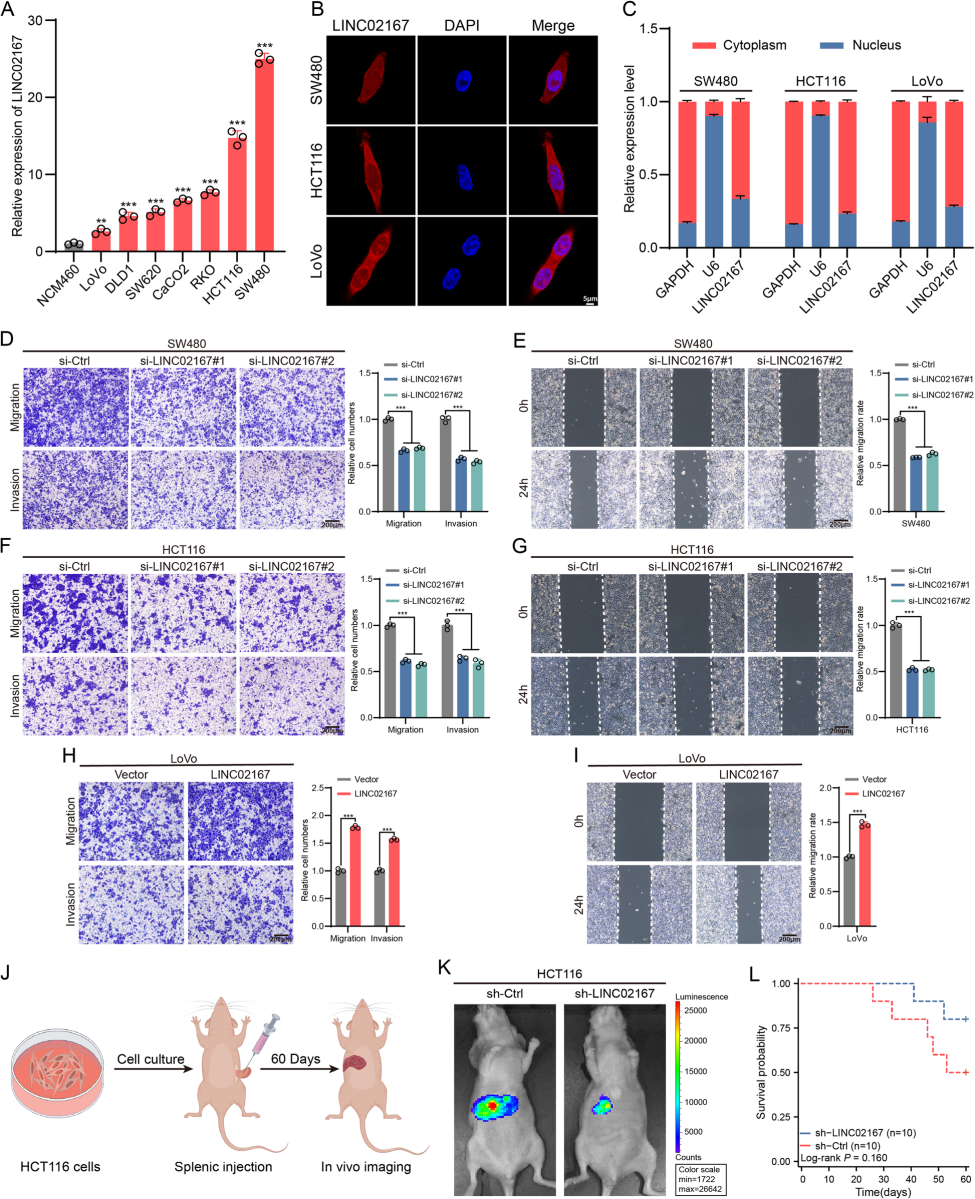
LINC02167 promotes CRC metastasis both in vitro and in vivo. **A** Relative expression levels of LINC02167 in the normal human intestinal epithelial cell line (NCM460) and CRC cell lines (HCT116, SW480, DLD1, SW620, CaCO2, LoVo, and RKO). **B**, **C** Subcellular localization of LINC02167 in CRC cells detected by FISH (B) and nuclear-cytoplasmic fractionation assay (**C**). **D**-**G** Effects of LINC02167 knockdown on migration and invasion in SW480 and HCT116 cells, as assessed by Transwell assays (**D**, **F**) and wound healing assays (**E**, **G**). **H**, **I** Effects of LINC02167 overexpression on migration and invasion in LoVo cells, analyzed through Transwell assays (**H**) and wound healing assays (**I**). **J** Schematic diagram of the CRC liver metastasis model in nude mice following splenic injection (By Figdraw). **K** Representative in vivo imaging and luminescence intensity of liver metastatic mice in the control group and LINC02167 knockdown group. **L** Survival curves of nude mice in the control group and LINC02167 knockdown group after splenic injection (*n* = 10), determined by the Kaplan–Meier method. *P < 0.05, **P < 0.01, ***P < 0.001

To investigate the functional impact and specific mechanisms of LINC02167 in CRC cells, we first analyzed its expression levels across various CRC cell lines using qRT-PCR. The results revealed that LINC02167 is significantly upregulated in HCT116, SW480, DLD1, SW620, RKO, CaCO2, and LoVo CRC cells compared to normal human colorectal epithelial cells (NCM460) (Fig. 2A). Recognizing the importance of subcellular localization in understanding lncRNA regulatory functions [25], we conducted FISH staining, which revealed a predominant cytoplasmic localization of LINC02167 in CRC cells (Fig. 2B). This finding was corroborated by cell fractionation qPCR and subcellular RNA analysis (Fig. 2C).

Among the cell lines, LINC02167 expression was highest in SW480 and HCT116 cells, while LoVo cells exhibited relatively low levels. To elucidate its biological role, we designed two siRNAs (si-LINC02167#1 and si-LINC02167#2), an shRNA (sh-LINC02167), and an overexpression plasmid (LINC02167). Knockdown experiments in SW480 and HCT116 cells, as well as overexpression in LoVo cells, were validated using qRT-PCR (Fig. S1A). Given the clinical association of LINC02167 with advanced CRC metastasis, we focused on its role in regulating CRC cell migration and invasion. Transwell and wound-healing assays demonstrated that LINC02167 knockdown significantly inhibited the migratory and invasive abilities of HCT116 and SW480 cells (Fig. 2D-G). Conversely, overexpression of LINC02167 enhanced migration and invasion in LoVo cells (Fig. 2H-I). These findings suggest that LINC02167 promotes CRC cell migration and invasion in vitro, highlighting its critical role in CRC metastasis.

To further validate the role of LINC02167 in CRC metastasis, we established a nude mouse liver metastasis model and conducted in vivo experiments using HCT116 cells (Fig. 2J). The in vivo imaging of mice was used to assess the formation of liver metastases in the model, and the results showed that knockdown of LINC02167 significantly inhibited the formation of liver metastases in the mice (Fig. 2K). Further, Kaplan–Meier survival analysis was conducted to evaluate the survival of the two groups of mice (Fig. 2L). Although statistical significance was not reached, the survival rate of the LINC02167 knockdown group was clearly superior to that of the control group. These results suggest that LINC02167 may promote the metastasis of CRC cells in vivo.

### LINC02167 promotes CRC metastasis by regulating the ERK/MAPK signaling pathway

**Fig. 3:**
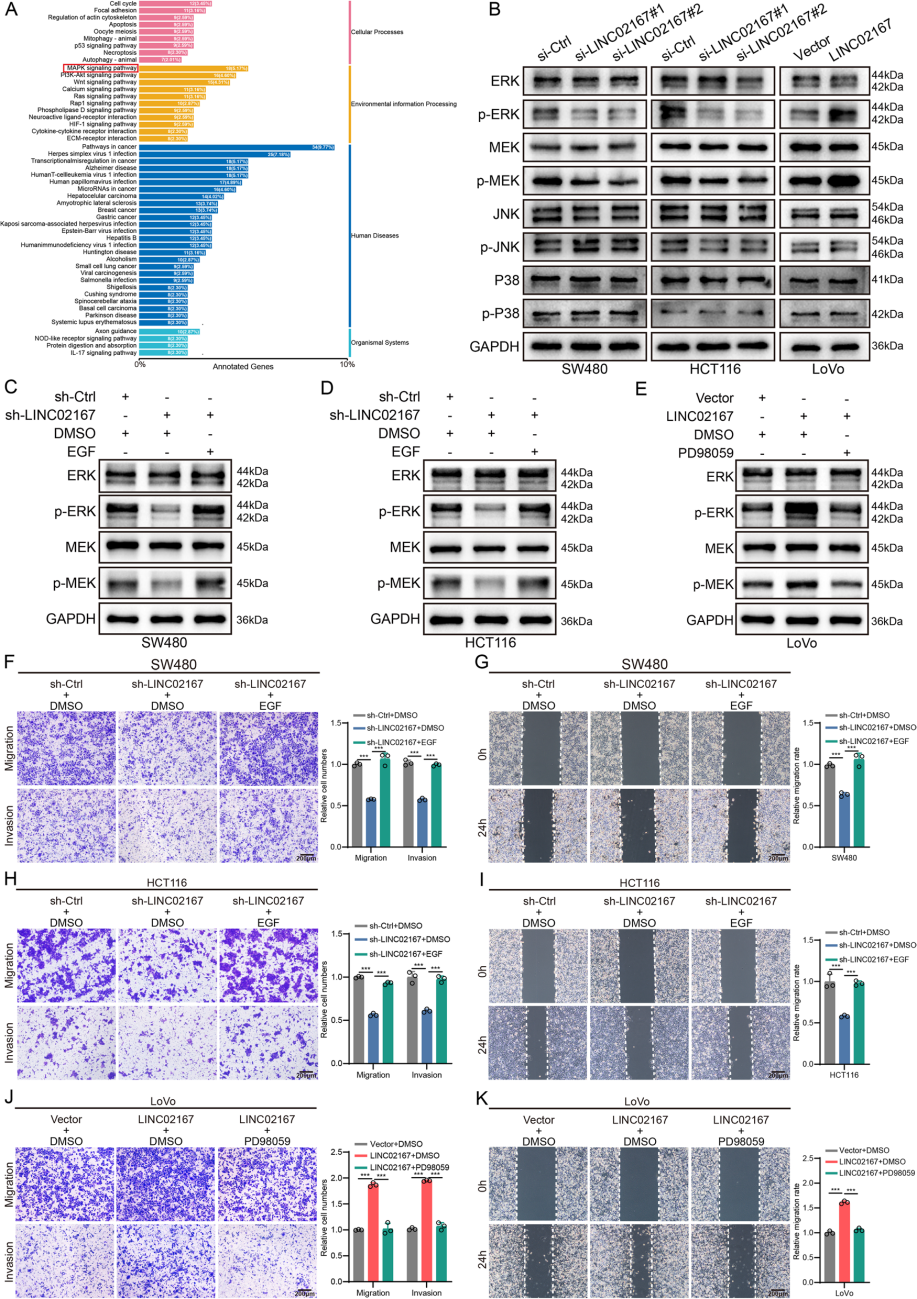
LINC02167 promotes CRC metastasis by regulating the ERK/MAPK signaling pathway. **A** KEGG pathway annotation analysis identifying signaling pathways associated with LINC02167. **B** Western blot analysis showing the effects of LINC02167 knockdown or overexpression on the expression of MAPK signaling pathway-related proteins in CRC cells. **C**, **D** Western blot detects the rescuing effect of EGF on the ERK/MAPK signaling pathway in LINC02167 knockdown CRC cells. **E** Western blot detects the inhibitory effect of PD98059 on the ERK/MAPK signaling pathway in LINC02167 overexpressing CRC cells. **F**-**I** Transwell assays (**F**, **H**) and wound healing assays (**G**, **I**) demonstrating that the inhibitory effect of LINC02167 knockdown on the migration and invasion abilities of HCT116 and SW480 cells is reversed by EGF treatment. **J**, **K** Transwell assays (**J**) and wound healing assays (**K**) indicating that the promotive effect of LINC02167 overexpression on migration and invasion in LoVo cells is reversed by PD98058 treatment. *P < 0.05, **P < 0.01, ***P < 0.001

To investigate the biological pathways through which LINC02167 CRC metastasis, we performed RNA-seq on CRC cells transfected with either LINC02167-specific siRNA or a control siRNA. This analysis identified a total of 946 differentially expressed genes (DEGs) (*P* < 0.05, |log_2_FC|≥ 0.585), with 489 genes upregulated and 457 downregulated. A volcano plot was used to illustrate the overall distribution of these DEGs (Fig. S1B), and clustering analysis was performed to determine gene expression patterns under different conditions (Fig. S1C). KEGG classification annotation results indicated that the differentially expressed genes were enriched in several cancer-related signaling pathways, such as MAPK, PI3K/AKT, and Wnt (Fig. 3A), suggesting that LINC02167 may promote CRC metastasis by regulating these cancer pathways.

To further explore whether LINC02167 has a regulatory effect on these cancer-related signaling pathways, we performed Western blot experiments to examine the changes in the levels of key proteins involved in the MAPK, PI3K/AKT, and Wnt signaling pathways after knockdown or overexpression of LINC02167. The results showed that LINC02167 had the most significant effect on the expression of phosphorylated ERK (p-ERK) and phosphorylated MEK (p-MEK) in the MAPK signaling pathway (Fig. 3B). While it also had some effect on the expression of phosphorylated PI3K (p-PI3K), phosphorylated AKT (p-AKT), and CTNNB1 protein in the PI3K/AKT and Wnt pathways, the effects were weaker (Fig. S1E-F). Moreover, no significant impact was observed on the expression of other key proteins in these three pathways (ERK, MEK, P38, p-P38, JNK, p-JNK, PI3K, and AKT). Therefore, we focused on the ERK/MAPK pathway. Previous studies have also reported that the ERK/MAPK signaling pathway plays an important role in promoting CRC metastasis [26, 27]. Therefore, we hypothesize that LINC02167 may promote CRC metastasis by regulating the ERK/MAPK signaling pathway.

To verify whether LINC02167 promotes CRC metastasis through the regulation of the ERK/MAPK pathway, we used the ERK/MAPK pathway activator EGF to enhance the activity of the ERK/MAPK pathway in LINC02167 knockdown CRC cells and the ERK/MAPK pathway inhibitor PD98059 to reduce the activity of the ERK/MAPK pathway in LINC02167 overexpressing CRC cells. Western blot experiments validated the effects of EGF and PD98059 on the activity of the ERK/MAPK pathway (Fig. 3C-E). Transwell and wound-healing assays revealed that EGF rescued the inhibitory effects of LINC02167 knockdown on cell migration and invasion in SW480 and HCT116 cells (Fig. 3F-I). Conversely, PD98059 attenuated the enhanced migratory and invasive capabilities induced by LINC02167 overexpression in LoVo cells (Fig. 3J-K). These results confirm that LINC02167 drives CRC cell metastasis by regulating the ERK/MAPK signaling pathway.

### KSR1 is a downstream target of LINC02167 in regulating the ERK/MAPK signaling pathway

**Fig. 4:**
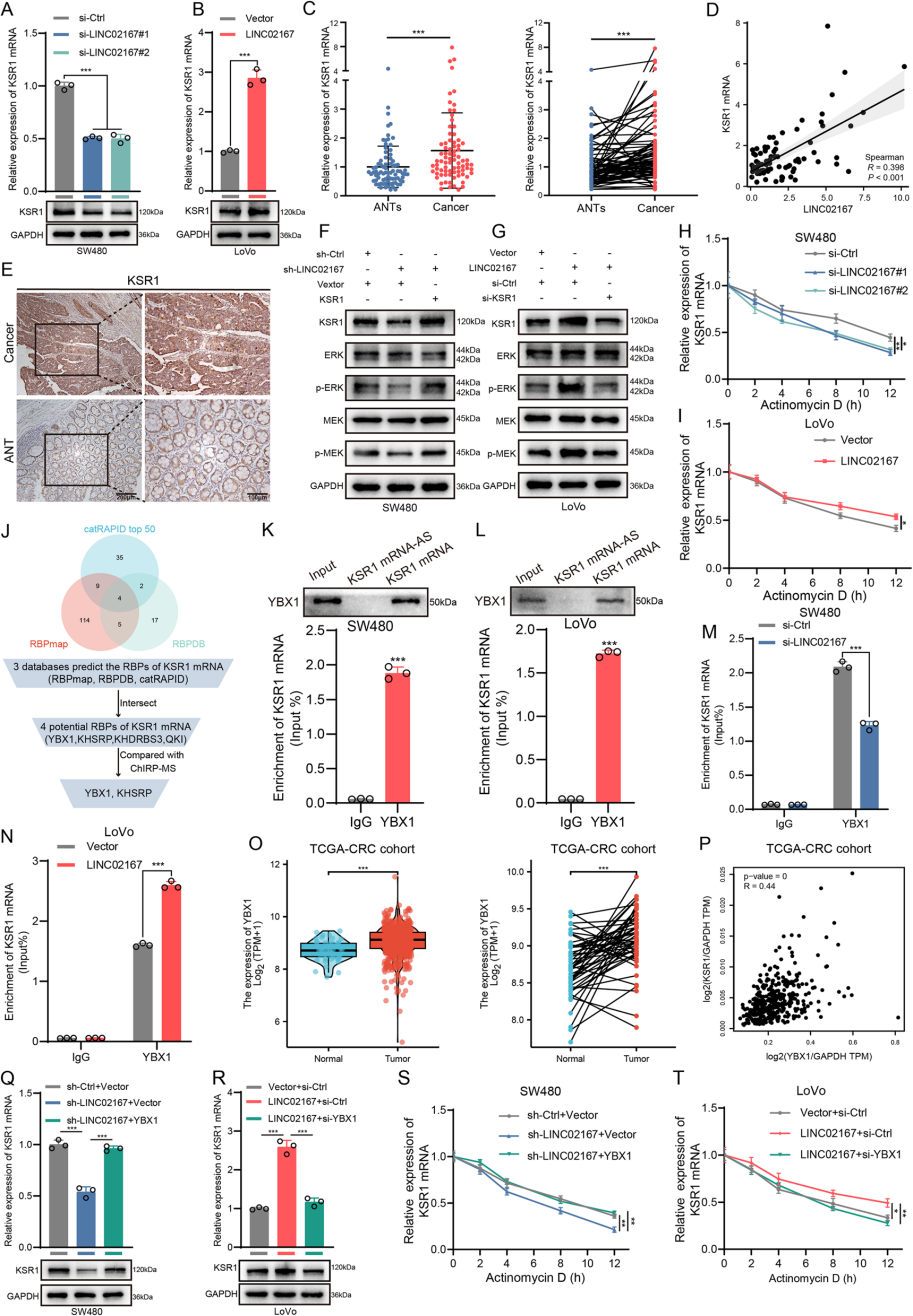
LINC02167 interacts with YBX1 to stabilize KSR1 mRNA and regulate the ERK/MAPK signaling pathway. **A** Analysis of KSR1 expression changes following LINC02167 knockdown in SW480 cells. **B** Analysis of KSR1 expression changes following LINC02167 overexpression in LoVo cells. **C** qRT-PCR analysis of KSR1 mRNA expression in CRC tissues and paired ANTs from clinical samples (*n* = 80). **D** Correlation analysis of LINC02167 and KSR1 mRNA expression in clinical samples. **E** IHC analysis of KSR1 expression in CRC tissues and paired ANT. **F** Western blot analysis showing that KSR1 overexpression reverses the reduction in p-ERK and p-MEK levels caused by LINC02167 knockdown in SW480 cells. **G** Western blot analysis showing that KSR1 knockdown reverses the increase in p-ERK and p-MEK levels caused by LINC02167 overexpression in LoVo cells. **H** RNA stability analysis showing decreased KSR1 mRNA stability following LINC02167 knockdown in SW480 cells. **I** RNA stability analysis showing increased KSR1 mRNA stability following LINC02167 overexpression in LoVo cells. **J** Schematic diagram of the screening process for LINC02167-binding proteins. **K**, **L** Biotin-labeled RNA pull-down and RIP experiments showing the interaction between YBX1 and KSR1 mRNA in SW480 and LoVo cells. **M** RIP analysis showing reduced KSR1 mRNA enrichment in YBX1 immunoprecipitates following LINC02167 knockdown in SW480 cells. **N** RIP analysis showing increased KSR1 mRNA enrichment in YBX1 immunoprecipitates following LINC02167 overexpression in LoVo cells. **O** TCGA database analysis of YBX1 mRNA expression in CRC tissues compared to normal tissues. **P** Correlation analysis of YBX1 and KSR1 expression in CRC tissues based on TCGA data. **Q** Analysis of the effect of YBX1 overexpression on KSR1 mRNA and protein expression in LINC02167-knockdown SW480 cells. **R** Analysis of the effect of YBX1 knockdown on KSR1 mRNA and protein expression in LINC02167-overexpressing LoVo cells. **S** RNA stability analysis showing that YBX1 overexpression rescues the reduction in KSR1 mRNA stability caused by LINC02167 knockdown in SW480 cells. **T** RNA stability analysis showing that YBX1 knockdown reverses the increase in KSR1 mRNA stability caused by LINC02167 overexpression in LoVo cells. *P < 0.05, **P < 0.01, ***P < 0.001

To investigate the specific mechanism by which LINC02167 regulates the ERK/MAPK signaling pathway, we further examined the expression changes of several key ERK/MAPK signaling pathway regulatory genes in CRC cells with stable knockdown of LINC02167 via qRT-PCR. Notably, the scaffold protein KSR1, which sustains the RAF-MEK-ERK signaling cascade [23, 24], was most significantly downregulated in LINC02167-knockdowned CRC cells (Fig. S2A-B). qRT-PCR and Western blot analyses confirmed that LINC02167 significantly modulates both mRNA and protein levels of KSR1 in CRC cells (Fig. 4A-B; Fig. S2C). Subsequently, we assessed KSR1 mRNA expression in CRC tissue samples from our clinical cohort using qRT-PCR, revealing a significant upregulation of KSR1 mRNA in CRC tissues (Fig. 4C). Correlation analysis showed a strong positive association between KSR1 mRNA and LINC02167 expression (Fig. 4D). IHC staining (Fig. 4E) and TCGA database analysis (Fig. S2D) further validated the upregulation of KSR1 in CRC tissues. These results suggest that KSR1 may be a downstream target of LINC02167 in regulating the ERK/MAPK signaling pathway in CRC.

To further elucidate the role of KSR1 in regulating the ERK/MAPK signaling pathway and CRC cell migration and invasion, we constructed siRNA targeting KSR1 (si-KSR1) and overexpression plasmids (KSR1). Western blot analyses showed that the activity of the ERK/MAPK signaling pathway is directly influenced by KSR1 levels (Fig. S2E). Moreover, in LINC02167-knockdown HCT116 and SW480 cells, overexpression of KSR1 rescued the effects of LINC02167 knockdown on ERK/MAPK signaling pathway activity (Fig. 4F; Fig. S2F) and cell migration and invasion (Fig. S3A-D). Conversely, KSR1 knockdown in LoVo cells reversed the increased ERK/MAPK signaling pathway activation (Fig. 4G) and enhanced cell migration and invasion (Fig. S3E-F) observed with LINC02167 overexpression. Interestingly, treatment with Actinomycin D or DRB to inhibit mRNA synthesis revealed that LINC02167 regulates the stability of KSR1 mRNA. Specifically, KSR1 mRNA stability was significantly reduced in LINC02167-knockdown CRC cells (Fig. 4H; Fig. S2G-I), while it was notably enhanced in LINC02167-overexpressing cells (Fig. 4I; Fig. S2J), suggesting that LINC02167 influences KSR1 expression through modulation of its mRNA stability. These findings suggest that LINC02167 promotes CRC metastasis by regulating the ERK/MAPK signaling pathway through modulation of KSR1 mRNA stability.

### LINC02167 interacts with YBX1 to regulate KSR1 mRNA stability

Previous studies have suggested that lncRNAs typically function in the cytoplasm by interacting with RNA-binding proteins (RBPs) [28]. Consistent with this, our GO molecular function (GO-MF) enrichment analysis revealed that the most significantly enriched term was "binding" (Fig. S1D), suggesting that LINC02167 may regulate KSR1 mRNA stability through interactions with specific proteins. To identify potential binding partners for LINC02167, we performed ChIRP assays using a commercially synthesized LINC02167 probe, followed by MS analysis. According to the criteria of Score Sequest HT > 10 and Unique Peptides ≥ 2, we selected a total of 482 high-confidence RBPs of LINC02167 from the MS results. Additionally, we used online databases to predict potential RBPs of KSR1 mRNA. By intersecting predictions from RBPDB, RBPmap, and the top 50 ranked RBPs from catRAPID, we identified 4 potential RBPs for KSR1 mRNA. Comparing these with the MS results for LINC02167, we identified 2 RBPs that bind both LINC02167 and KSR1 mRNA (Fig. 4J). Upon further literature review, we identified YBX1 as a candidate RNA/DNA-binding protein that regulates the stability of target mRNAs, influencing processes such as apoptosis, proliferation, differentiation, and drug resistance in cancer [29]. Additionally, YBX1 has also been reported as a key regulator of the MEK/ERK pathway-dependent gene signature in CRC cells [30]. Consequently, we focused on YBX1 for further investigation. We first validated the interaction between YBX1 and KSR1 mRNA in CRC cells using RNA pull-down and RIP assays (Fig. 4K-L). Notably, RIP experiments revealed that knockdown of LINC02167 reduced the enrichment of KSR1 mRNA in YBX1 immunoprecipitates (Fig. 4M), while overexpression of LINC02167 significantly increased this enrichment (Fig. 4N), suggesting that LINC02167 influences the interaction between YBX1 and KSR1 mRNA.

Analysis of the TCGA database confirmed that YBX1 is significantly upregulated in CRC (Fig. 4O) and its expression is positively correlated with KSR1 expression (Fig. 4P). To further validate the role of YBX1 in regulating KSR1 expression and CRC metastasis, we constructed YBX1 siRNA (si-YBX1) and overexpression plasmids (YBX1) (Fig. S4A). qRT-PCR and Western blot analyses demonstrated that LINC02167 and YBX1 jointly regulate KSR1 expression (Fig. 4Q-R; Fig. S4B). RNA stability assays revealed that YBX1 overexpression restored KSR1 mRNA stability impaired by LINC02167 knockdown (Fig. 4S; Fig. S4C-E), while YBX1 knockdown reversed the enhanced stability of KSR1 mRNA induced by LINC02167 overexpression (Fig. 4T; Fig. S4F). These findings suggest that LINC02167 and YBX1 collaboratively regulate KSR1 mRNA stability. Functional rescue experiments showed that YBX1 overexpression restored the migratory and invasive abilities of HCT116 and SW480 cells impaired by LINC02167 knockdown (Fig. S5A-D). Conversely, YBX1 knockdown reversed the enhanced migratory and invasive capabilities observed in LINC02167-overexpressing LoVo cells (Fig. S5E-F). Collectively, these findings demonstrate that LINC02167 drives CRC metastasis by directly interacting with YBX1 to stabilize KSR1 mRNA.

**Fig. 5:**
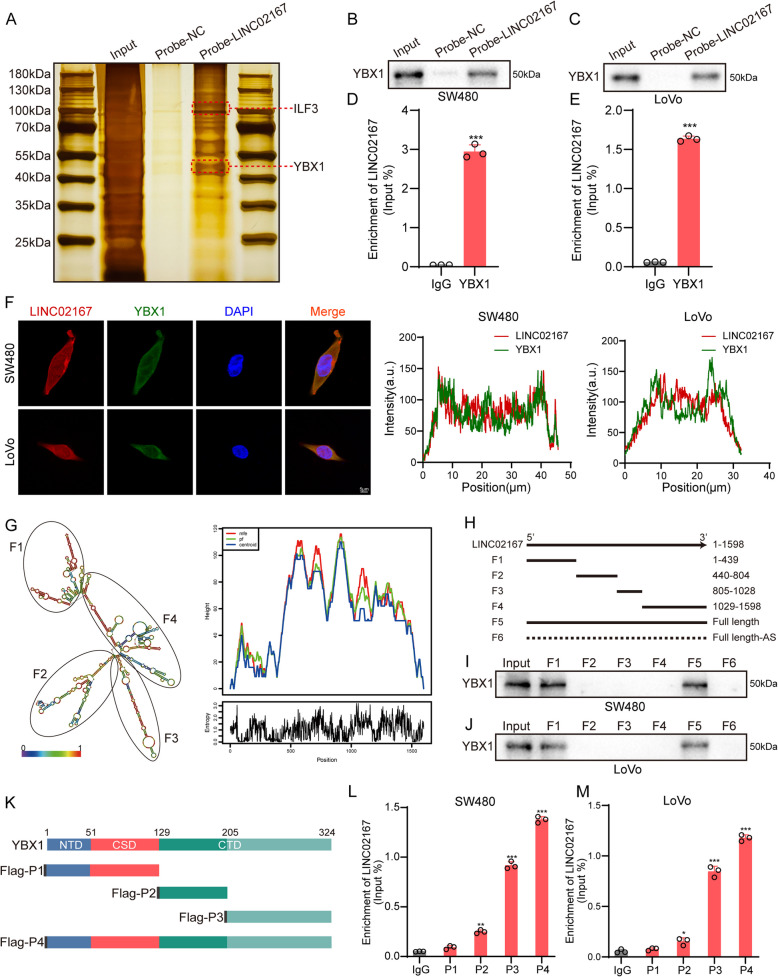
Characterization of the interaction between LINC02167 and YBX1. **A** Silver staining image of chromatin isolation by RNA purification (CHIRP) assay using LINC02167 probe in SW480 cells. **B**, **C** Western blot analysis showing YBX1 expression in samples bound to the LINC02167 probe in CRC cells. **D**, **E** RIP-qPCR analysis of LINC02167 enrichment in YBX1 antibody-bound complexes in CRC cells. **F** Confocal microscopy images showing colocalization of LINC02167 (red) and YBX1 (green) in cells. Nuclei are stained with DAPI (blue). **G** Predicted secondary structure of LINC02167 generated using RNAfold. **H** Schematic representation of truncated LINC02167 constructs. **I**, **J** Western blot analysis showing YBX1 levels in samples pulled down by different biotin-labeled truncated LINC02167 constructs (F1-F4), full-length LINC02167 (F5), and antisense full-length LINC02167 (F6) in CRC cells. **K** Schematic of Flag-tagged YBX1 truncations (P1-P3) and full-length YBX1 (P4) constructs. **L**, **M** RIP-qPCR analysis of LINC02167 enrichment in different Flag-tagged YBX1 truncations and full-length YBX1 in CRC cells. *P < 0.05, **P < 0.01, ***P < 0.001

Next, we further confirmed the interaction between LINC02167 and YBX1 and explored the specific region of their interaction. First, the silver staining of the proteins bound by LINC02167's ChIRP probes showed a distinct specific band at approximately 50 kDa (Fig. 5A). Further Western blot analysis revealed that YBX1 could be detected in the proteins that bound to the LINC02167 probe (Fig. 5B-C). Additionally, RIP-qPCR results showed significant enrichment of LINC02167 in the immunoprecipitates of YBX1 (Fig. 5D-E). These results indicate that an interaction exists between LINC02167 and YBX1 in CRC cells. FISH and IF co-localization experiments further demonstrated that LINC02167 and YBX1 co-localize in CRC cells (Fig. 5F; Fig. S6A), providing additional evidence for their interaction. Interestingly, Western blot analysis showed that neither knockdown nor overexpression of LINC02167 affected YBX1 expression levels in CRC cells (Fig. S4G), indicating that LINC02167 does not regulate YBX1 expression. Although secondary structures are not always conserved, they remain a good indicator for identifying functional elements in lncRNAs. To determine the secondary structure of LINC02167 that mediates its interaction with YBX1, we used the RNAfold online tool to predict the secondary structure of LINC02167 (Fig. 5G). The prediction revealed that LINC02167 consists of four major substructures. We then constructed biotinylated LINC02167 fragments based on its predicted secondary structure: F1 (1–439 nt), F2 (440–804 nt), F3 (805–1028 nt), and F4 (1029–1598 nt), along with a full-length biotinylated probe (F5) and an antisense probe (F6), for RNA pull-down assays (Fig. 5H). Western blot analysis of the pull-down proteins revealed that YBX1 was only detectable in the F1 and F5 samples (Fig. 5I-J), indicating that the 1–439 nt region of LINC02167 is crucial for its interaction with YBX1. YBX1 contains three structural domains: the N-terminal domain (NTD), cold shock domain (CSD), and C-terminal domain (CTD) [31]. To identify the domain of YBX1 involved in the interaction with LINC02167, we cloned a series of Flag-tagged YBX1 truncation plasmids: P1 (1–129 aa), P2 (130–205 aa), P3 (206–324 aa), and P4 (1–324 aa) (Fig. 5K; Fig. S6B). RIP-qPCR results showed that the CTD of YBX1 is essential for binding to LINC02167 (Fig. 5L-M). These findings are consistent with predictions from the catRAPID database (Fig. S6C) and our MS analysis (Fig. S6D). Therefore, the 1–439 nt region of LINC02167 and the CTD domain of YBX1 are essential for their interaction.

### m^5^C modification mediates YBX1-dependent stabilization of KSR1 mRNA

**Fig. 6:**
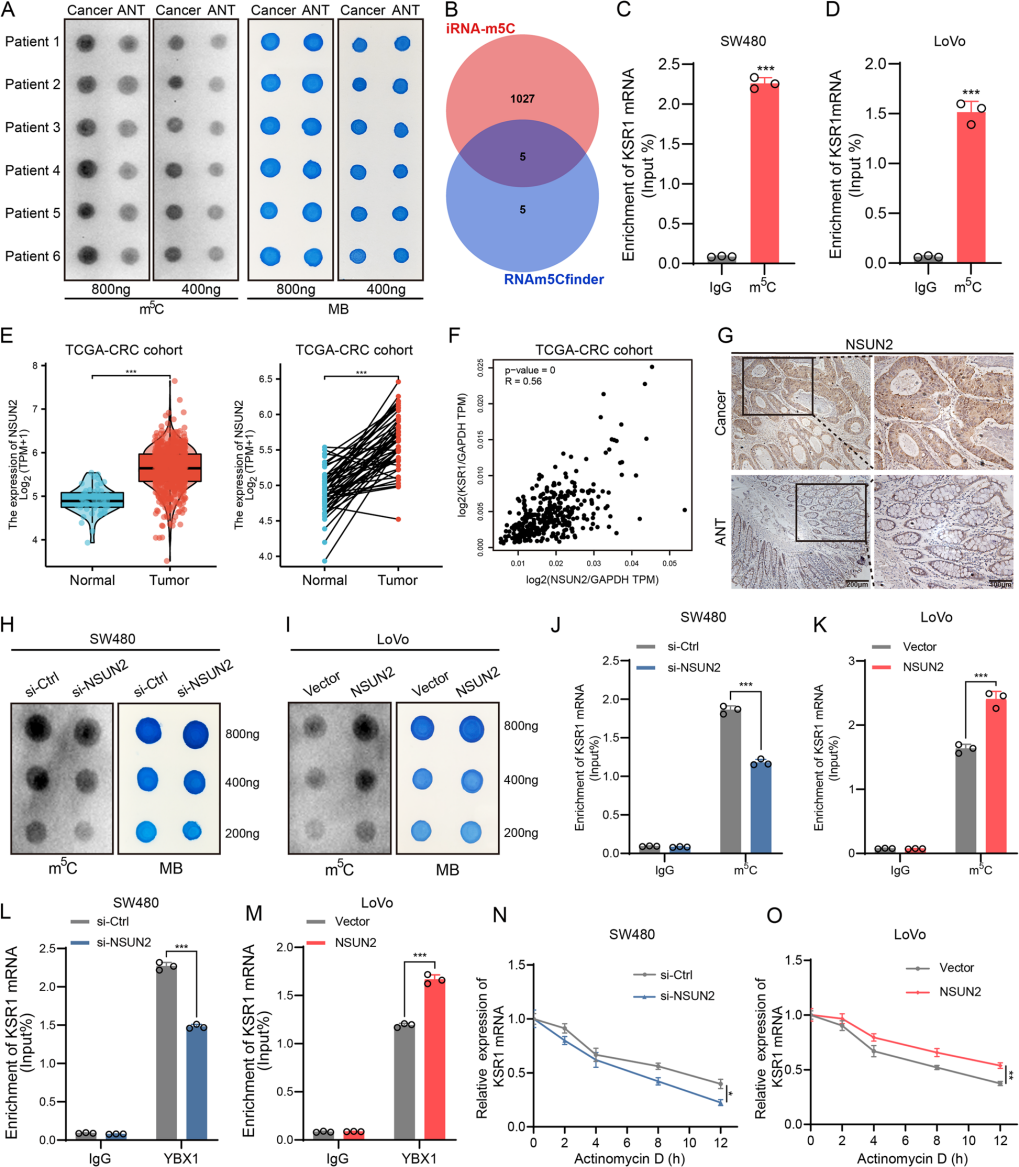
m^5^C modification mediates YBX1-dependent stabilization of KSR1 mRNA. **A** Dot blot analysis of m^5^C levels in CRC tissues and paired ANTs. **B** Predicted m^5^C modification sites on KSR1 mRNA using iRNA-m5C and RNAm5Cfinder. **C**, **D** MeRIP-qPCR analysis of m^5^C levels in KSR1 mRNA in CRC cells. **E** Analysis of NSUN2 mRNA expression in CRC and normal tissues from the TCGA database. **F** Correlation analysis between NSUN2 and KSR1 expression in CRC tissues from the TCGA database. **G** IHC analysis of NSUN2 expression in CRC tissues and paired ANT. **H**, **I** Dot blot analysis of m^5^C level changes in CRC cells with NSUN2 knockdown or overexpression. **J**, **K** MeRIP-qPCR analysis of m^5^C levels in KSR1 mRNA following NSUN2 knockdown or overexpression in CRC cells. **L**, **M** RIP-qPCR analysis of KSR1 mRNA enrichment in YBX1 immunoprecipitates following NSUN2 knockdown or overexpression in CRC cells. **N**, **O** RNA stability analysis showing the impact of NSUN2 knockdown or overexpression on KSR1 mRNA stability in CRC cells. *P < 0.05, **P < 0.01, ***P < 0.001

It is well-established that YBX1 is an m^5^C reader protein capable of recognizing m^5^C-modified mRNAs to maintain their stability [11]. Therefore, we hypothesized that m^5^C modification might mediate the role of YBX1 in regulating KSR1 mRNA stability. Recent studies have demonstrated elevated m^5^C levels in CRC, which promote CRC progression [12]. We verified that m^5^C levels are significantly higher in CRC tissues compared to matched normal tissues through Dot Blot analysis (Fig. 6A), which underscores the potential role of m^5^C in CRC pathogenesis. Using RNAm5Cfinder and iRNA-m5C prediction tools, we identified potential m^5^C modification sites on KSR1 mRNA (Fig. 6B). Consistent with this prediction, MeRIP-qPCR confirmed m^5^C modifications on KSR1 mRNA in CRC cells (Fig. 6C-D), raising the possibility that YBX1 may bind these m^5^C sites to stabilize KSR1 mRNA. To understand the mechanism behind KSR1 mRNA’s m^5^C modification, we examined the expression of 11 reported m^5^C methyltransferases in CRC tissues using TCGA data [32]. NSUN2 emerged as the most highly expressed methyltransferase in CRC, with a significant expression differential between CRC and normal tissues (Fig. 6E; Fig. S7A). Correlation analysis revealed a positive association between NSUN2 and KSR1 expression (Fig. 6F), which was further supported by IHC staining showing high NSUN2 levels in CRC tissues (Fig. 6G). Thus, NSUN2 was selected for further investigation. To confirm NSUN2's role, we transfected CRC cells with NSUN2-targeted siRNA (si-NSUN2) and an NSUN2 overexpression plasmid (NSUN2) (Fig. S7B). Dot Blot results showed that NSUN2 knockdown reduced global m^5^C levels in CRC cells (Fig. 6H), and MeRIP-qPCR confirmed a specific reduction in KSR1 mRNA’s m^5^C levels (Fig. 6J). Conversely, NSUN2 overexpression increased m^5^C levels globally (Fig. 6I) and on KSR1 mRNA (Fig. 6K). Importantly, RIP analysis revealed that NSUN2 knockdown weakened, whereas NSUN2 overexpression strengthened, the interaction between YBX1 and KSR1 mRNA (Fig. 6L-M). RNA stability assays further demonstrated that NSUN2 knockdown diminished KSR1 mRNA stability (Fig. 6N; Fig. S7C), whereas NSUN2 overexpression enhanced it (Fig. 6O; Fig. S7D). These findings indicate that NSUN2-mediated m^5^C methylation facilitates YBX1-dependent stabilization of KSR1 mRNA in an m^5^C-dependent manner.

### YBX1 and ILF3 cooperatively maintain KSR1 mRNA stability via LINC02167

**Fig. 7:**
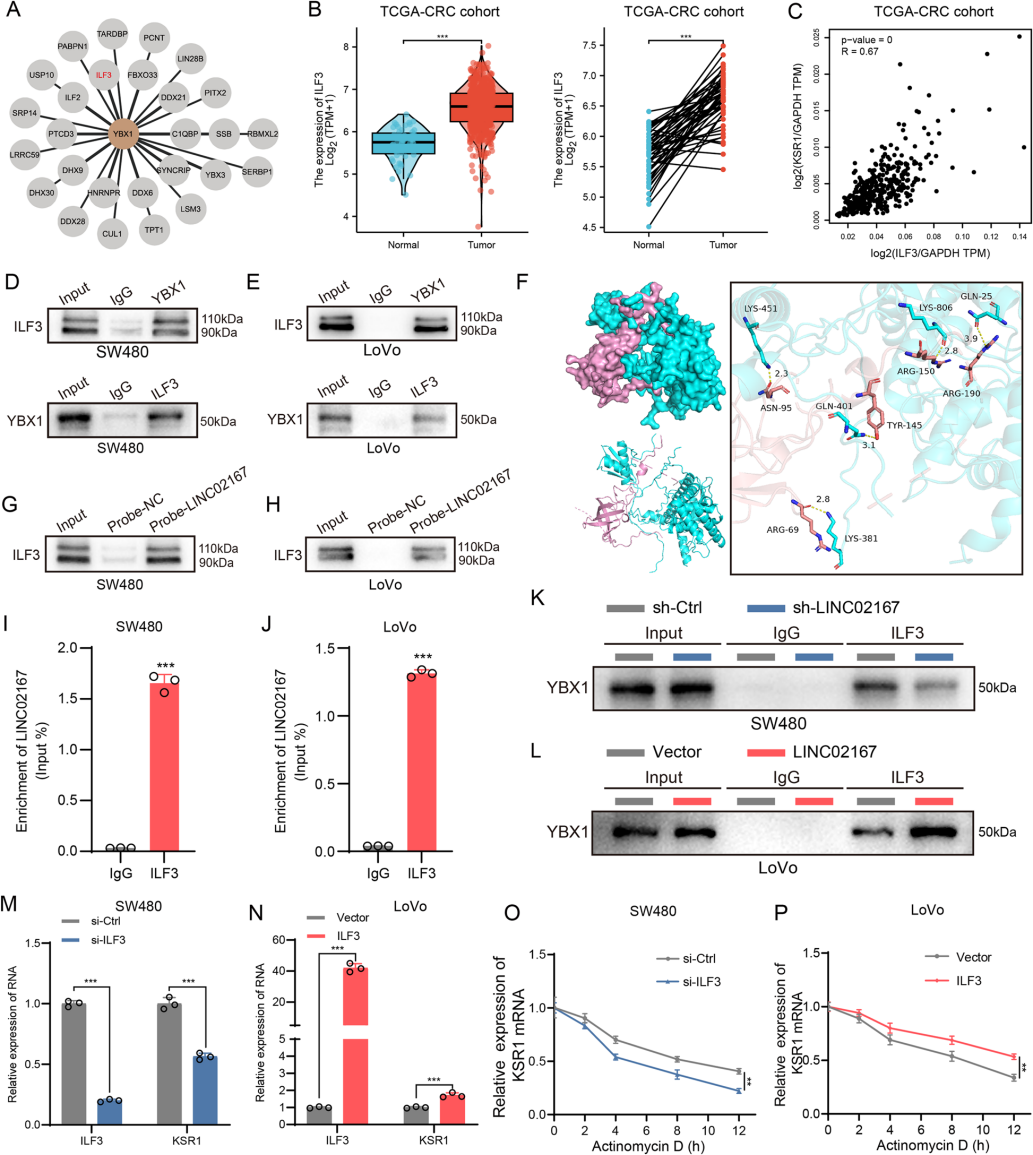
YBX1 and ILF3 cooperatively maintain KSR1 mRNA stability via LINC02167. **A** Prediction of proteins interacting with YBX1 from the HUMAN PROTEIN ATLAS website. **B** Analysis of ILF3 mRNA expression in CRC tissues and normal tissues using the TCGA database. **C** Correlation analysis between ILF3 and KSR1 expression in CRC tissues from the TCGA database. **D**, **E** Co-immunoprecipitation (Co-IP) assays demonstrating the interaction between YBX1 and ILF3 in CRC cells. **F** Molecular docking predicts the binding mode between YBX1 (pink) and ILF3 (cyan) and identifies the interacting amino acids. Specifically, ASN95, ARG69, TYR145, ARG150, and ARG190 of YBX1 form five hydrogen bonds with LYS451, LYS381, GLN401, LYS806, and GLN25 of ILF3, respectively. **G**, **H** Western blot analysis of ILF3 expression in samples bound to the LINC02167 probe in CRC cells. **I**, **J** RIP-qPCR analysis showing enrichment of LINC02167 in ILF3 antibody-bound complexes in CRC cells. **K**, **L** Western blot analysis of YBX1 co-precipitated with ILF3 in CRC cells after LINC02167 knockdown or overexpression. **M**, **N** qRT-PCR analysis of KSR1 mRNA expression in CRC cells after ILF3 knockdown or overexpression. **O**, **P** RNA stability analysis of KSR1 mRNA stability in CRC cells after ILF3 knockdown or overexpression

Previous studies have shown that YBX1 often functions in coordination with one or more proteins during the regulation of specific mRNA stability [31]. Thus, we explored whether additional proteins are involved in the LINC02167/YBX1-mediated regulation of KSR1 mRNA stability. Using the Human Protein Atlas database, we identified interleukin enhancer-binding factor 3 (ILF3) as a potential binding partner of YBX1 (Fig. 7A). Notably, ILF3 has been reported to synergize with YBX1 in stabilizing NANOG mRNA [33]. TCGA database analysis revealed that ILF3 is significantly upregulated in CRC tissues (Fig. 7B) and positively correlates with KSR1 expression (Fig. 7C), suggesting its potential role in regulating KSR1 expression and CRC progression. Interestingly, ILF3 was also identified as a LINC02167-binding protein in our ChIRP-MS analysis (Fig. 5A), further supporting its involvement in this regulatory network.

Accumulating evidence suggests that lncRNAs can act as scaffolds to facilitate interactions between binding proteins [34, 35]. We hypothesized that LINC02167 might function as a scaffold to enhance the interaction between YBX1 and ILF3, thereby stabilizing KSR1 mRNA. To test this hypothesis, we performed Co-IP assays and confirmed that YBX1 interacts with ILF3 in CRC cells (Fig. 7D-E). Molecular docking analysis revealed a direct interaction between YBX1 and ILF3, with a docking score of −240.95 kcal/mol. This interaction is mediated by five hydrogen bonds, formed between key residues of YBX1 (ASN-95, ARG-69, TYR-145, ARG-150, and ARG-190) and ILF3 (LYS-451, LYS-381, GLN-401, LYS-806, and GLN-25) (Fig. 7F). Additionally, ChIRP and RIP assays demonstrated that ILF3 interacts with LINC02167 (Fig. 7G-J), confirming the formation of a LINC02167/YBX1/ILF3 complex. Next, we examined whether LINC02167 influences the interaction between YBX1 and ILF3 in CRC cells. Co-IP experiments showed that knockdown of LINC02167 reduced the amount of YBX1 co-precipitated with ILF3 compared to the control group (Fig. 7K). Conversely, overexpression of LINC02167 increased the co-precipitation of YBX1 with ILF3 (Fig. 7L).

To further validate the role of ILF3 in maintaining KSR1 mRNA stability, we constructed siRNA targeting ILF3 (si-ILF3) and ILF3 overexpression plasmids (ILF3) (Fig. S7E). qRT-PCR and RNA stability assays showed that ILF3 knockdown significantly decreased KSR1 mRNA expression (Fig. 7M) and stability (Fig. 7O; Fig. S7F) compared to the control group. Conversely, ILF3 overexpression elevated KSR1 mRNA levels (Fig. 7N) and enhanced its stability (Fig. 7P; Fig. S7G). Additionally, Western blot results indicated that neither knockdown nor overexpression of LINC02167 affected ILF3 protein levels in CRC cells, suggesting that LINC02167 interacts with ILF3 without regulating its expression (Figure. S7H). These results confirm that LINC02167 acts as a molecular scaffold to promote the interaction between ILF3 and YBX1, collaboratively maintaining KSR1 mRNA stability in CRC cells.

### MYC transcriptionally activates LINC02167 expression in CRC

The mechanism underlying the upregulation of LINC02167 in CRC remains unclear. Previous studies have reported that transcription factors (TFs) can enhance lncRNA expression by regulating the transcription of their host genes [36, 37]. Using the UCSC Genome Browser in conjunction with JASPAR, we identified several potential transcription factors that may bind to the promoter region of LINC02167 and regulate its transcription (Fig. S7J). Among these candidates, MYC stood out due to its well-documented role in CRC. MYC has been shown to interact with lncRNAs in CRC, influencing CRC progression by promoting or repressing the transcription of specific lncRNAs [38].

**Fig. 8:**
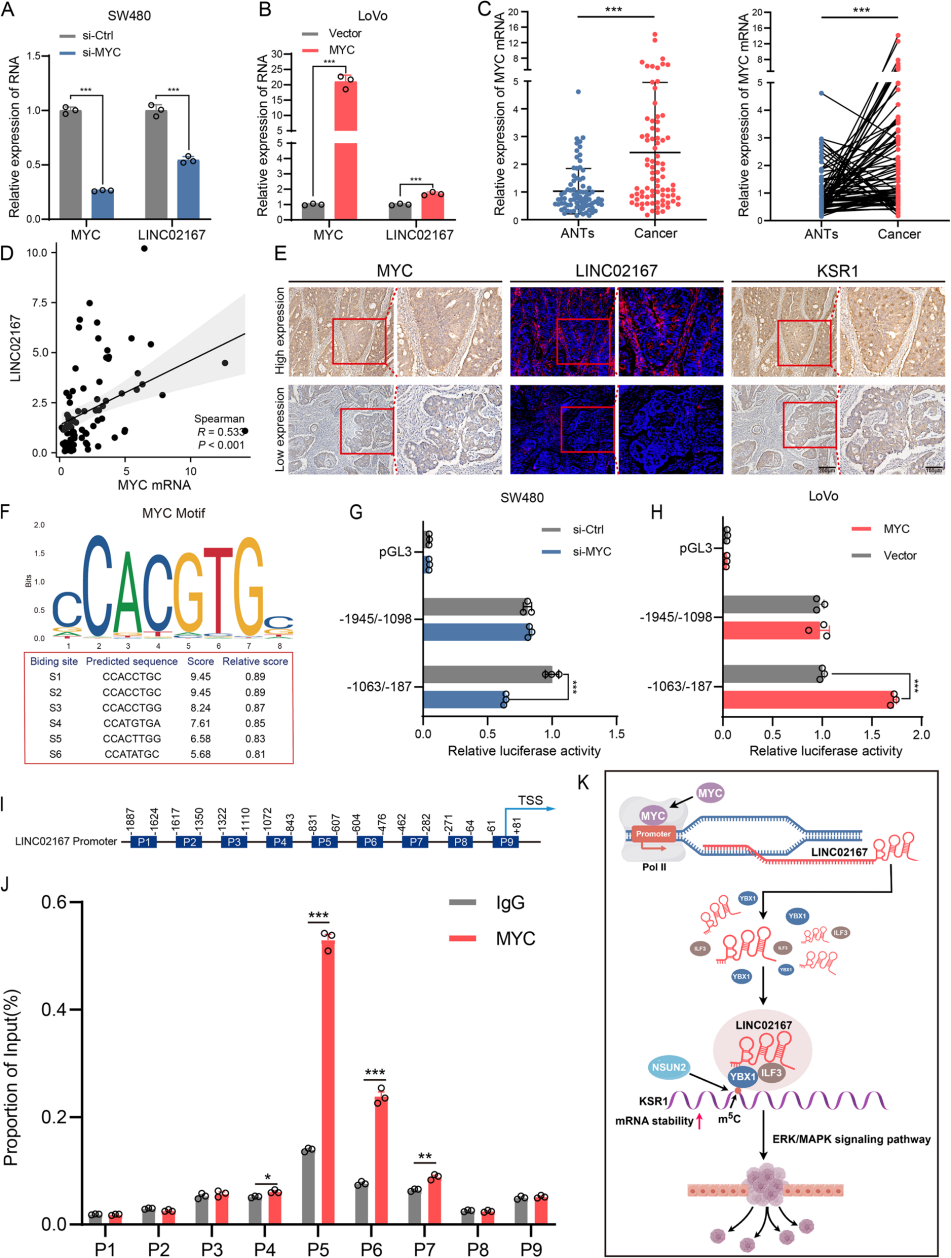
MYC transcriptionally activates LINC02167 expression in CRC. **A** Analysis of LINC02167 expression changes following MYC knockdown in SW480 cells. **B** Analysis of LINC02167 expression changes following MYC overexpression in LoVo cells. **C** qRT-PCR analysis of MYC expression in CRC tissues and paired ANTs from clinical samples (*n* = 80). **D** Correlation analysis between MYC and LINC02167 expression in clinical samples. **E** Representative IHC and FISH images showing MYC, LINC02167, and KSR1 expression in tumors with high and low MYC expression. **F** Prediction of potential YBX1 binding sites in the LINC02167 promoter region using the JASPAR website. **G**, **H** Luciferase reporter assay showing that luciferase activity driven by the LINC02167 promoter region fragment (− 1063 to − 187) is significantly reduced in SW480 cells with MYC knockdown, while luciferase activity is increased in LoVo cells with MYC overexpression. **I** Schematic representation of the LINC02167 promoter region. **J** ChIP-qPCR analysis showing enrichment of different regions of the LINC02167 promoter in MYC antibody-bound complexes in SW480 cells, with normal rabbit IgG antibody as a negative control. **K** Schematic illustration of the mechanism by which LINC02167 is upregulated in CRC and stabilizes KSR1 mRNA in an m^5^C-dependent manner, activating the ERK/MAPK signaling pathway to promote colorectal cancer metastasis (By Figdraw). *P < 0.05, **P < 0.01, ***P < 0.001

Analysis of the TCGA database revealed that MYC is also highly expressed in CRC tissues (Fig. S7K). To investigate the impact of MYC on LINC02167 expression in CRC cells, we constructed and transfected MYC-specific siRNA (si-MYC) and a MYC overexpression plasmid (MYC) (Fig. S7I). qRT-PCR analysis showed that LINC02167 expression was significantly downregulated upon si-MYC transfection (Fig. 8A; Fig. S7L), while it was upregulated following MYC overexpression (Fig. 8B). Additionally, we validated MYC expression in a cohort of 80 paired CRC patient samples using qRT-PCR. The results confirm that MYC is significantly upregulated in CRC tissues (Fig. 8C) and positively correlates with LINC02167 expression (Fig. 8D). Furthermore, IHC and FISH staining of pathological sections from CRC tissues further confirmed that LINC02167 expression is elevated in MYC-high CRC tissues, accompanied by increased KSR1 expression. Conversely, in MYC-low CRC tissues, the expression levels of both LINC02167 and KSR1 are reduced (Fig. 8E).

Using JASPAR, we identified six high-confidence MYC binding sites within the LINC02167 promoter, designated as S1 to S6 (Fig. 8F). To evaluate whether MYC transcriptionally activates LINC02167, we cloned two different fragments of the LINC02167 promoter and inserted them into luciferase reporter gene vectors. Luciferase assays showed that the − 1063/ − 187 fragment, but not the − 1945/ − 1098 fragment, was responsive to changes in MYC expression. Specifically, MYC knockdown reduced luciferase activity driven by the − 1063/ − 187 fragment (Fig. 8G; Fig. S7M), whereas MYC overexpression enhanced it (Fig. 8H), confirming that MYC-responsive elements are located within this region. To investigate the binding sites of MYC within the LINC02167 promoter, nine primer pairs (P1–P9) were designed to target distinct segments of this promoter region. (Fig. 8I). ChIP-qPCR analysis further demonstrated that MYC directly binds to the P4–P7 regions of the LINC02167 promoter (Fig. 8J). Notably, the predicted binding sites S1, S3, and S6, as identified through JASPAR, were located within these regions of the promoter. These findings collectively suggest that the transcription factor MYC mediates the upregulation of LINC02167 expression in CRC.

## Discussion

This study reveals the regulatory role of LINC02167 in CRC metastasis, highlighting its upstream influence on the ERK/MAPK signaling pathway through its interaction with YBX1 in an m^5^C dependent manner. This novel finding not only enhances our understanding of ERK/MAPK signaling but also offers new insights into the potential of targeting this pathway in CRC treatment.

The ERK/MAPK signaling pathway, as a central signaling pathway in cancer progression, is involved in key processes such as cell proliferation, survival, and migration [16]. Although targeted therapies against KRAS, MEK, and RAF inhibitors have shown promise clinically, they often face challenges with limited efficacy and drug resistance [39, 40]. For example, when treating BRAF-mutant solid tumors, cancer cells often activate alternative pathways like PI3K/AKT or re-activate ERK signaling to bypass inhibitor effects [41]. Such bypass activation is particularly prevalent in CRC, making it essential to explore new methods for upstream regulation of the ERK/MAPK signaling pathway, which could help to overcome the limitations of current therapies and improve efficacy. Our study shows that LINC02167 indirectly modulates ERK/MAPK signaling pathway activity by stabilizing KSR1 mRNA. Notably, as a scaffold protein, KSR1 plays a crucial role in maintaining specificity and fidelity of signal transduction in the ERK/MAPK cascade. Therefore, manipulating LINC02167 expression may effectively regulate ERK/MAPK signaling pathway activity upstream, potentially avoiding drug resistance issues caused by direct kinase inhibition. This finding provides a theoretical basis for targeting upstream lncRNAs in regulating the ERK/MAPK signaling pathway, and subsequent studies could further assess its therapeutic potential.

In this mechanism, YBX1 acts as a binding partner of LINC02167, playing a critical role. Previous research has established that YBX1 frequently functions as a binding protein for various lncRNAs, contributing to the progression of multiple cancers, including CRC. For instance, our previous study reported that lncRNA POU6F2-AS1 binds to YBX1 to promote transcriptional activation, leading to CRC cell proliferation [42]. In another study, lncRNA MILIP was shown to bind YBX1, affecting Snail translation and promoting renal clear cell carcinoma metastasis [43]. YBX1 is also well-known for its role in maintaining mRNA stability of target genes. For example, YBX1 influences the stability of SKIL [14] and NRF2 [44] mRNAs in CRC. Our research finds that LINC02167 binds to the CTD domain of YBX1, jointly stabilizing KSR1 mRNA as a downstream target. By modulating LINC02167/YBX1 interaction, it may be possible to alter KSR1 expression, indirectly regulating ERK/MAPK signaling pathway function. Future research could explore small molecule inhibitors targeting the LINC02167/YBX1 axis to test its indirect regulation of the ERK/MAPK signaling pathway.

m^5^C modification is a key form of RNA epigenetics that significantly impacts mRNA stability, transcription, and translation. This study is the first to reveal how m^5^C modification influences the ERK/MAPK signaling pathway by affecting the interaction between lncRNA and proteins. We found that NSUN2 facilitates YBX1 binding to KSR1 mRNA via m^5^C modification, thereby enhancing KSR1 stability. NSUN2, an m^5^C methyltransferase, has been shown to play a significant role in cancer progression in various cancers, including lung, hepatocellular, and breast cancers [45–47]. Compared to existing research, our findings provide new evidence on the regulatory mechanism of m^5^C modification in CRC metastasis and lay the groundwork for developing m^5^C -based targeted therapies. However, the specific m^5^C modification sites on KSR1 mRNA remain unidentified, limiting our comprehensive understanding of its regulatory mechanism.

Though m^5^C -targeted therapies are in the early stages, studies have already explored treatments targeting other epigenetic modifications. For example, Decitabine has been successfully used to inhibit DNA methyltransferase for treating hematological malignancies [48, 49]. Building on the success of such epigenetic-targeted strategies, future research could explore inhibitors of NSUN2 or other m^5^C methyltransferases as potential treatments for metastatic CRC. Given NSUN2’s role in regulating m^5^C modification of KSR1 mRNA, this direction provides a translational pathway for m^5^C modification applications. Further, m^5^C modification could serve as a biomarker to identify patients resistant to current therapies, thereby aiding in personalized treatment strategy development.

Our research confirms that ILF3, as an RNA-binding protein, plays a synergistic role in the LINC02167/YBX1 complex by increasing the half-life of KSR1 mRNA, supporting the sustained invasiveness of CRC cells. ILF3 is known to stabilize mRNAs of proteins like Cyclin E1, ERp57, and SGOC, which promote the proliferation and metastasis of cancers, including hepatocellular, renal, and CRC [50–52]. Although ILF3’s role in other cancers has been validated, its function in CRC has not been systematically studied. Clinically, targeting this complex could involve disrupting LINC02167/YBX1 or YBX1/ILF3 interactions to reduce the survival capacity of metastatic cancer cells, thereby enhancing the effectiveness of existing treatments. However, the precise interaction sites between ILF3, YBX1, and LINC02167 remain unclear, limiting our complete understanding of this complex. Future research should further investigate these interactions to deepen our understanding of their role in CRC metastasis.

Beyond these regulatory mechanisms, our study reveals that LINC02167 upregulation is directly controlled by MYC, further establishing LINC02167 as a downstream effector of MYC. MYC is a classic oncogene and transcription factor that regulates thousands of cancer-related genes involved in CRC processes such as proliferation, metastasis, metabolism, immune modulation, and biosynthesis [53–56]. Our findings demonstrate that MYC transcriptionally activates LINC02167, directly driving CRC progression. This new MYC-LINC02167 axis not only enhances our understanding of MYC’s role in cancer metastasis but also provides an alternative approach for MYC-targeted therapy by indirectly reducing MYC’s oncogenic effects through LINC02167 inhibition. MYC has long been recognized as an undruggable target due to its complex biological functions and structural characteristics [57], making LINC02167 inhibition a potentially feasible alternative strategy, particularly for CRC patients with high MYC expression.

From a clinical translation perspective, RNA interference (RNAi) technology offers the possibility of developing therapeutic strategies targeting LINC02167. For instance, antisense oligonucleotides (ASO) and liposome/nanoparticle-delivered siRNA provide the potential for directly targeting LINC02167. By designing precise molecules to reduce or inhibit the expression of specific lncRNAs, this approach can influence cancer progression. On the other hand, small molecule strategies offer an alternative therapeutic route by disrupting the interactions between lncRNA and its molecular partners, or by directly intervening in the structure of lncRNAs, thereby modulating their function [58, 59]. Currently, a phase I trial is underway for oligonucleotide therapy targeting the long non-coding RNA TUG1 in recurrent glioblastoma, which provides a direct reference for targeting LINC02167 in therapy [60]. In addition, CRISPR/Cas9 gene editing technology, based on nanoparticle delivery systems, can specifically regulate this lncRNA by epigenetically silencing the promoter region of LINC02167 [61]. Regarding the molecular mechanism discovered in this study, where LINC02167 interacts with the YBX1 protein, the development of specific inhibitors targeting this complex may offer a novel therapeutic strategy. For example, small molecule inhibitors can be developed through virtual screening or structure-based drug design methods [62]. These inhibitors would target the CTD domain involved in the binding between LINC02167 and YBX1, disrupting their spatial conformation or competitively binding to key sites, thus blocking their interaction while avoiding interference with other physiological functions of YBX1. Recent studies have shown that small molecule inhibitors that block certain sequences where HOTAIR interacts with PRC2 or LSD1, as well as the interactions between PRC2 and LSD1, can inhibit breast cancer cell metastasis [59]. Future research could verify the effectiveness of these strategies using organoid models (PDO) and patient-derived xenograft (PDX) models under conditions simulating the tumor microenvironment, which would accelerate their clinical translation process.

The clinical relevance of LINC02167 lies not only in its potential as a therapeutic target but also in its significant association with poor prognosis. Our study found that high LINC02167 expression in CRC patients was associated with shorter OS and DFS. This indicates that LINC02167 could be a potential prognostic marker. High LINC02167 expression is linked to increased metastasis risk, and it may serve as a clinical indicator to predict treatment efficacy, helping clinicians develop better therapeutic strategies for patients with possible resistance to ERK/MAPK-targeted therapy.

However, our study’s results are primarily based on in vitro experiments, and although we observed LINC02167’s inhibitory effects on CRC metastasis in mouse models, its specific associations with the ERK/MAPK signaling pathway and m^5^C modifications require validation in more complex in vivo settings. Additionally, while this study focuses mainly on the LINC02167/KSR1 axis, there may be other downstream target genes and signaling pathways regulated by this lncRNA complex that remain to be discovered. Future studies could employ whole-transcriptome sequencing or genomic screening to comprehensively analyze LINC02167’s target gene network, providing a deeper understanding of its multifaceted role in CRC.

In conclusion, LINC02167’s upstream regulatory role in the ERK/MAPK signaling pathway, coupled with its interaction with m^5^C modification, offers a new perspective for CRC treatment. Alongside current targeted therapies and epigenetic modification studies, our findings pave the way for exploring LINC02167 and associated molecules as therapeutic targets in CRC. By studying the LINC02167/YBX1/KSR1 axis and m^5^C modification mechanisms in depth, this research provides a foundation for developing more precise and effective therapeutic strategies in the future. However, further in vivo research and clinical validation are necessary to facilitate the personalized treatment of metastatic CRC.

## Conclusion

Our study identifies LINC02167 as a critical regulator of CRC metastasis and elucidates its mechanistic role as a molecular scaffold that stabilizes KSR1 mRNA in an m^5^C-dependent manner by enhancing the interaction between YBX1 and ILF3. This stabilization promotes the activation of the ERK/MAPK signaling pathway, a key driver of CRC progression. Additionally, MYC-mediated transcriptional upregulation of LINC02167 underscores its central role in CRC pathogenesis. These findings advance our understanding of the molecular basis of CRC metastasis and highlight LINC02167 as a promising therapeutic target. Future efforts to disrupt the LINC02167/YBX1/KSR1 axis may offer new strategies for combating CRC metastasis and improving clinical outcomes.

## Supplementary Information

Additional file 1. Table S1. Relationship between LINC02167 expression and clinicopathological features in TMAs cohort. Table S2. The sequences of oligonucleotides and probes used in this study. Table S3. The sequences of primers used for qRT–PCR

Additional file 2. Supplemental Materials and Methods

Additional file 3. Fig. S1. (A) Validation of LINC02167 knockdown or overexpression efficiency in CRC cells. (B) Volcano plot showing gene expression differences between control and LINC02167 knockdown groups in CRC cells. (C) Heatmap of differentially expressed genes between control and LINC02167 knockdown groups. (D) Gene Ontology (GO) analysis, including biological process (BP), cellular component (CC), and molecular function (MF), highlighting significantly enriched pathways after LINC02167 knockdown. (E) Western blot analysis showing the effects of LINC02167 knockdown or overexpression on the expression of Wnt signaling pathway-related proteins in CRC cells. (F) Western blot analysis showing the effects of LINC02167 knockdown or overexpression on the expression of PI3K/AKT signaling pathway-related proteins in CRC cells. *P < 0.05, **P < 0.01, ***P < 0.001.

Additional file 4. Fig. S2 (A, B) qRT-PCR analysis of the expression changes in key ERK/MAPK regulatory genes in SW480 and HCT116 cells with stable LINC02167 knockdown. (C) Analysis of KSR1 expression changes after LINC02167 knockdown in HCT116 cells. (D) TCGA database analysis of KSR1 expression in CRC tissues compared to normal tissues. (E) Western blot analysis showing the effect of KSR1 knockdown or overexpression on ERK/MAPK signaling pathway activity. (F) Western blot analysis showing that KSR1 overexpression rescues the reduction in p-ERK and p-MEK levels caused by LINC02167 knockdown in HCT116 cells. (G-I) RNA stability assay showing reduced KSR1 mRNA stability after LINC02167 knockdown in CRC cells. (J) RNA stability analysis showing increased KSR1 mRNA stability following LINC02167 overexpression in LoVo cells. *P < 0.05, **P < 0.01, ***P < 0.001.

Additional file 5. Fig. S3 (A-D) Transwell assays (A, C) and wound healing assays (B, D) showing that the suppression of CRC cell migration and invasion by LINC02167 knockdown is reversed by KSR1 overexpression. (E, F) Transwell assay (E) and wound healing assay (F) showing that the promotion of CRC cell migration and invasion by LINC02167 overexpression is reversed by KSR1 knockdown. *P < 0.05, **P < 0.01, ***P < 0.001.

Additional file 6. Fig. S4 (A) Validation of YBX1 knockdown or overexpression efficiency in CRC cells. (B) Analysis of the effect of YBX1 overexpression on KSR1 mRNA and protein expression following LINC02167 knockdown in HCT116 cells. (C-E) RNA stability assay showing that YBX1 overexpression rescues the reduction in KSR1 mRNA stability caused by LINC02167 knockdown in CRC cells. (F) RNA stability analysis showing that YBX1 knockdown reverses the increase in KSR1 mRNA stability caused by LINC02167 overexpression in LoVo cells. (G) Analysis of the effect of LINC02167 knockdown or overexpression on YBX1 protein levels in CRC cells. *P < 0.05, **P < 0.01, ***P < 0.001.

Additional file 7. Fig. S5 (A-D) Transwell assays (A, C) and wound healing assays (B, D) showing that the suppression of CRC cell migration and invasion caused by LINC02167 knockdown is reversed by YBX1 overexpression. (E, F) Transwell assay (E) and wound healing assay (F) showing that the promotion of CRC cell migration and invasion caused by LINC02167 overexpression is reversed by YBX1 knockdown. *P < 0.05, **P < 0.01, ***P < 0.001.

Additional file 8. Fig. S6 (A) Co-localization of LINC02167 (red) and YBX1 (green) in HCT116 cells detected by confocal microscopy. Nuclei were stained with DAPI (blue). (B) Western blot analysis of Flag-tagged YBX1 constructs (P1: 1–129 aa, P2: 130–205 aa, P3: 206–324 aa) transfected into SW480 cells. (C) Predicted interaction regions between LINC02167 and YBX1 based on catRAPID analysis. (D) Representative YBX1 peptides detected by mass spectrometry (MS) as binding to LINC02167

Additional file 9. Fig. S7 (A) Analysis of the expression levels of 11 m^5^C "Writers" in CRC tissues and normal tissues from the TCGA database. (B) Validation of NSUN2 knockdown or overexpression efficiency in CRC cells. (C, D) RNA stability analysis of KSR1 mRNA stability in CRC cells after NSUN2 knockdown or overexpression. (E) Validation of ILF3 knockdown or overexpression efficiency in CRC cells. (F, G) RNA stability analysis of KSR1 mRNA stability in CRC cells after ILF3 knockdown or overexpression. (H) Analysis of the effect of LINC02167 knockdown or overexpression on ILF3 protein levels in CRC cells. (I) Validation of MYC knockdown or overexpression efficiency in CRC cells. (J) Prediction of LINC02167 transcriptional regulators using the UCSC Genome Browser. (K) Analysis of MYC expression in CRC tissues compared to normal tissues from the TCGA database. (L) Analysis of LINC02167 expression changes after MYC knockdown in HCT116 cells. (M) Luciferase reporter assay showing that knockdown of MYC significantly reduces luciferase activity driven by the LINC02167 promoter (-1063 to -187 fragment) in HCT116 cells. *P < 0.05, **P < 0.01, ***P < 0.001.

## Data Availability

No datasets were generated or analysed during the current study.

## Abbreviations

CRC: Colorectal cancer
TCGA: The Cancer Genome Atlas
LncRNA: Long non-coding RNA
m^5^C: 5-Methylcytosine
KSR1: Kinase Suppressor of Ras 1
YBX1: Y-box binding protein 1
TMA: Tissue microarray
HE: Hematoxylin eosin
OS: Overall survival
DFS: Disease-free survival
KEGG: Kyoto encyclopedia of genes and genomes
GO: Gene Ontology
shRNA: Short hairpin RNA
siRNA: Small interfering RNA
ANTs: Adjacent noncancerous tissues
qRT–PCR: Real-time quantitative polymerase chain reaction
RBPs: RNA-binding proteins
FISH: RNA fluorescence in situ hybridization
IHC: Immunohistochemical
RIP: RNA immunoprecipitation
Me-RIP: Methylated RNA Immunoprecipitation
ChIP: Chromatin immunoprecipitation
MS: Mass spectrometry
IF: Immunofluorescence
ILF3: Interleukin Enhancer Binding Factor 3
Co-IP: Co-immunoprecipitation
