# Supplementary material for: LINC02167 stabilizes KSR1 mRNA in an m5C-dependent manner to regulate the ERK/MAPK signaling pathway and promotes colorectal cancer metastasis
Source: J Exp Clin Cancer Res. 2025 Apr 15;44:121. doi: 10.1186/s13046-025-03368-w (PMC11998267; doi:10.1186/s13046-025-03368-w)
Supplement: Supplementary file 1 — Additional file 1. Table S1. Relationship between LINC02167 expression and clinicopathological features in TMAs cohort. Table S2. The sequences of oligonucleotides and probes used in this study. Table S3. The sequences of primers used for qRT–PCR [file 13046_2025_3368_MOESM1_ESM.zip › Additional file 1/Table S1.docx]

| **Variables** | **Cases** | **LINC02167 expression** | | ***P* ^a^** |
| --- | --- | --- | --- | --- |
|  |  | **Low(n=57)** | **High(n=59)** |  |
| Age (years) |  |  | | 0.982 |
| ≤60 | 51 | 25 | 26 |  |
| >60 | 65 | 32 | 33 |  |
| Gender |  |  | | 0.362 |
| Male | 66 | 30 | 36 |  |
| Female | 50 | 27 | 23 |  |
| Tumor diameter (cm) |  |  | | 0.062 |
| ≤5 | 61 | 35 | 26 |  |
| >5 | 55 | 22 | 33 |  |
| Depth of invasion |  |  | | **0.029** |
| T1/T2 | 23 | 16 | 7 |  |
| T3/T4 | 93 | 41 | 52 |  |
| Lymph node metastasis |  |  | | **<0.001** |
| N0 | 63 | 44 | 19 |  |
| N1/N2 | 53 | 13 | 40 |  |
| Distant metastasis |  |  | | **0.040** |
| M0 | 110 | 57 | 53 |  |
| M1 | 6 | 0 | 6 |  |
| TNM stage |  |  | | **<0.001** |
| I-II | 62 | 43 | 19 |  |
| III-IV | 54 | 14 | 40 |  |
| Differentiation |  |  | | 0.055 |
| Middle/High | 82 | 45 | 37 |  |
| Poor | 34 | 12 | 22 |  |

**Table 1** Relationship between LINC02167 expression and clinicopathological features of CRC patients

*P*-value ^a^ was measured by Pearson’s Chi-Squared test.
