# Supplementary material for: LINC02167 stabilizes KSR1 mRNA in an m5C-dependent manner to regulate the ERK/MAPK signaling pathway and promotes colorectal cancer metastasis
Source: J Exp Clin Cancer Res. 2025 Apr 15;44:121. doi: 10.1186/s13046-025-03368-w (PMC11998267; doi:10.1186/s13046-025-03368-w)
Supplement: Supplementary file 2 — Additional file 2. Supplemental Materials and Methods [file 13046_2025_3368_MOESM2_ESM.docx]

**Supplemental materials and methods**

**Cell lines and culture**

The normal human colorectal epithelial cell line NCM460 was obtained from INCELL, while CRC cell lines HCT116, DLD1, LoVo, CaCO2, RKO, SW480, and SW620 were sourced from the Cell Bank of the Chinese Academy of Sciences (Shanghai, China). HCT116 cells were cultured in McCoy’s 5A medium with 10% fetal bovine serum (FBS) (Gibco, Cat# A5670701); DLD1, RKO, and LoVo cells in RPMI 1640 (Gibco, Cat# 11875093); NCM460 and SW480 cells in DMEM/high-glucose medium (Gibco, Cat# 11965092); and SW620 cells in L-15 medium (Gibco, Cat# 11415064). All cell lines were maintained at 37°C with 5% CO₂ and confirmed to be mycoplasma-free before use.

**Cell transfection**

siRNAs targeting LINC02167 (si-LINC02167#1, si-LINC02167#2), MYC (si-MYC), YBX1 (si-YBX1), ILF3 (si-ILF3), NSUN2 (si-NSUN2), and KSR1 (si-KSR1), as well as a non-targeting control (si-Ctrl), were designed and synthesized by Gene Pharma Technology (Shanghai, China). CRC cells were transfected with these siRNAs using jetPRIME® Polyplus Transfection (Polyplus-transfection S.A., Cat# 101000046) following the manufacturer’s protocol. Full-length sequences of LINC02167, MYC, YBX1, ILF3, NSUN2, and KSR1 were cloned into the pcDNA3.1 vector to construct overexpression plasmids, with the empty vector serving as a negative control. Transfections of these plasmids were performed using Hieff Trans™ Liposomal Transfection Reagent (Yeasen Biotechnology, Cat# 40802ES) according to the manufacturer’s instructions. siRNA and shRNA sequences are detailed in Additional file 1: Table S2.

**RNA extraction and qRT–PCR**

Total RNA was isolated from CRC specimens and cells using RNA Isolater Total RNA Extraction Reagent (Vazyme, Cat# R401-01) according to the manufacturer’s guidelines. The quality and concentration of the extracted samples were determined by a Nanodrop 2000 (Thermo Fisher Scientific). For qRT–PCR analysis of LINC02167 and mRNA expression, RNA was first reverse-transcribed to cDNA using HiScript II Q RT SuperMix (Vazyme, Cat# R223-01), which was then detected by ChamQ SYBR qPCR Master Mix (Vazyme, Cat# Q311-02) on a LightCycler 96 Instrument (Roche, Switzerland) using the following thermal conditions: 95 °C for 30 s; 40 cycles at 95 °C for 10 s and 60 °C for 60 s; and a melting curve analysis. The relative RNA expression level was calculated using the 2^-ΔΔCT^ method, GAPDH serving as a reference for RNAs. The sequences of the primers used are listed in Additional file 1: Table S3.

**Nuclear–cytoplasmic separation**

Nuclear and cytoplasmic RNA from CRC cells was isolated using the PARIS™ Kit (Thermo Fisher Scientific, Cat# AM1921) according to the manufacturer’s protocol. Briefly, CRC cells (5×10^6^ per sample) were harvested and incubated on ice for 10 minutes in cell fractionation buffer supplemented with an RNase inhibitor. The suspension was centrifuged at 4°C for 5 minutes at 500g, and the supernatant was collected as the cytoplasmic fraction for RNA extraction. The pellet, representing the nuclear fraction, was resuspended in cell disruption buffer and incubated on ice to extract nuclear RNA. The extracted RNA was reverse-transcribed and analyzed by qPCR. GAPDH was used as a cytoplasmic marker, while U6 served as a nuclear marker.

**Immunohistochemistry (IHC)** **staining**

Subcutaneous tumor specimens were fixed in 4% formalin, embedded in paraffin, and sectioned into 4-μm-thick slices. IHC was performed using an Ultrasensitive Two-Step Immunohistochemistry Detection Kit (Zhongshan Biotech, Cat# PV-6000) following the manufacturer’s protocol. The sections were incubated with primary antibodies against MYC (1:1000, Proteintech, Cat# 10828-1-AP, RRID: AB_2148585), KSR1 (1:200, Abcam, Cat# ab68483, RRID: AB_11157290), and NSUN2 (1:200, Proteintech, Cat# 20854-1-AP, RRID: AB_10693629). IHC staining images were captured using an Olympus microscope (Tokyo, Japan).

**Fluorescence *in situ* hybridization (FISH) staining**

The localization of LINC02167 in CRC cells and its expression in tissue sections were determined using a Ribo™ Fluorescent In Situ Hybridization Kit (RIBOBIO, Cat# C10910) following the manufacturer’s protocol. LINC02167 expression levels in tissue microarrays (TMAs) were assessed based on FISH staining. Staining intensity was scored as follows: 0, no staining; 1, low staining; and 2, high staining. The percentage of positively stained cells was graded as follows: 0, 0% (no stained cells); 1, 1–24%; 2, 25–49%; 3, 50–74%; and 4, 75–100%. The final score was calculated by multiplying the staining intensity score by the score for the percentage of positively stained cells. CRC samples in TMAs were then categorized into low-expression (score 0–3) and high-expression (score 4–8) groups.

**Immunofluorescence (IF) staining**

For IF analysis, CRC cells were seeded onto glass coverslips and cultured overnight. Cells were fixed with 4% paraformaldehyde, permeabilized with 0.2% Triton X-100, and blocked with BSA. Subsequently, cells were incubated with anti-YBX1 antibody (1:200, Proteintech, 20339-1-AP) at 4°C overnight, followed by incubation with a CoraLite488-conjugated goat anti-rabbit IgG (H+L) secondary antibody (1:500, Proteintech, Cat# SA00013-2, RRID: AB_2797132) at 37°C for 1 hour. Nuclei were stained with 4′,6-diamidino-2-phenylindole (DAPI). Representative images were acquired using a confocal laser scanning microscope (CLSM, Leica STELLARIS 5, Germany).

**Western blot**

Transfected CRC cells were lysed in RIPA buffer (Beyotime, Cat# P0013B) supplemented with protease and phosphatase inhibitors. Protein concentrations were determined using a BCA Protein Assay Kit (Beyotime, Cat# P0011). Equal amounts of protein were resolved by SDS-PAGE and transferred onto PVDF membranes (Millipore, Cat# IPVH00010). After blocking with 5% skimmed milk, the membranes were incubated overnight at 4°C with primary antibodies against MYC (1:10,000, Proteintech, Cat# 10828-1-AP, RRID: AB_2148585), ERK (1:1,000, Cell Signaling Technology, Cat# 4695, RRID:AB_390779), p-ERK (1:2,000, Cell Signaling Technology, Cat# 4370, RRID:AB_2315112), MEK (1:1,000, Cell Signaling Technology, Cat# 9122, RRID:AB_823567), p-MEK (1:1,000, Cell Signaling Technology, Cat# 9154, RRID:AB_2138017), JNK(1:1,000, Cell Signaling Technology, Cat# 9252, RRID:AB_2250373), p-JNK(1:1,000, Cell Signaling Technology, Cat# 4668, RRID:AB_823588), P38 (1:1,000, Bioss, Cat# bs-0637R, RRID:AB_10856281), p-P38 (1:1,000, Bioss, Cat# bs-0636R, RRID:AB_10856595), PI3K (1:1,000, Cell Signaling Technology, Cat# 4257, RRID:AB_659889), p-PI3K (1:1,000, Cell Signaling Technology, Cat# 17366, RRID:AB_2895293), AKT (1:1,000, Cell Signaling Technology, Cat# 9272, RRID:AB_329827), p-AKT (1:2,000, Cell Signaling Technology, Cat# 4060, RRID:AB_2315049), CTNNB1 (1:10,000, Proteintech, Cat# 51067-2-AP, RRID: AB_2086128), YBX1 (1:10,000, Proteintech Cat# 20339-1-AP, RRID:AB_10665424), ILF3 (1:5,000, Proteintech Cat# 19887-1-AP, RRID:AB_10666431), KSR1 (1:1,000, Abcam Cat# ab68483, RRID: AB_11157290), NSUN2 (1:5,000, Proteintech Cat# 20854-1-AP, RRID: AB_10693629), GAPDH (1:10,000, Proteintech Cat# 60004-1-Ig, RRID:AB_2107436), and Flag (1:10,000, Proteintech, Proteintech Cat# 66008-4-Ig, RRID:AB_2918475). The following day, membranes were incubated with the appropriate secondary antibodies for 2 hours at room temperature, followed by three washes with TBST buffer. Protein signals were visualized using Chemistar™ High-sig ECL Western Blot Substrate (Tanon, Shanghai, China).

**RNA immunoprecipitation (RIP), methylated RNA immunoprecipitation (MeRIP)**

RIP was performed with a Magna RNA-Binding Protein Immunoprecipitation Kit (Millipore, Cat# 17-700) according to the manufacturer’s instructions. Briefly, transfected cells were lysed on ice for 15 min with RIP Lysis Buffer containing protease inhibitor cocktail and RNase inhibitor, and then centrifuged to collect the supernatant. The beads-antibody complex was obtained by incubating 5 μg of anti-YBX1 antibody, anti-ILF3 antibody, or normal rabbit IgG and magnetic beads protein A/G with rotation for 30 min at room temperature. Next, the cell lysate supernatant and the complex prepared above were rotationally incubated at 4°C overnight. Each immunoprecipitant flag was resuspended in proteinase K buffer and incubated at 55 °C for 30 min. Finally, RNA was extracted by phenol, chloroform and isoamyl alcohol according to the manufacturer’s instructions, and was detected by qRT-PCR. Similarly, MeRIP assays were performed using anti-m^5^C antibodies (Abcam, Cat# ab10805, RRID: AB_442823), and the RNA content in the immunoprecipitated complexes was examined by qRT-PCR.

**Co-immunoprecipitation (Co-IP) assay**

For the Co-IP assay, cells were lysed in IP buffer supplemented with protease inhibitors and incubated with antibodies against YBX1, ILF3, or normal rabbit IgG at 4°C overnight. Protein A/G agarose beads (MCE, Cat# HY-K0202) were added and incubated at 4°C for an additional 2 hours. The bead-protein complexes were washed three times with lysis buffer, and the eluted proteins were analyzed by Western blot using antibodies specific to YBX1 and ILF3.

**RNA sequencing**

Total RNA was extracted from vector control and LINC02167-overexpressing CRC cells using Trizol reagent (Vazyme, Cat# R401-01). RNA quantity and quality were assessed with a NanoDrop 2000 spectrophotometer (Thermo Fisher Scientific, MA, USA). Library preparation and transcriptome sequencing were carried out by Biomarker Technologies (Beijing, China). DEGs were identified based on the criteria of |log_2_FC| ≥ 0.585 and *P* < 0.05.

**Comprehensive identification of RNA-Binding proteins by mass spectrometry (ChIRP-MS)**

The antisense oligonucleotide ChIRP probes targeting LINC02167 were designed and synthesized by RIBOBIO (Guangzhou, China). CRC cells (2 × 10⁷ per sample) were collected and lysed in lysis buffer as instructed by the manufacturer. ChIRP probes were added to the lysates, and the mixture was incubated overnight at 4°C. Streptavidin agarose resin (Thermo Fisher Scientific, Cat# 20347) were subsequently added and incubated for 2 hours. After washing, the beads were eluted, and the protein samples were denatured and resolved by SDS-PAGE. Proteins were visualized using a silver staining kit (Sangon Biotech, Cat# [C510027-0010](https://store.sangon.com/productDetail?productInfo.code=C510027)), and differentially stained bands were excised and subjected to MS analysis (LC-Bio Technology, Hangzhou, China).

**Molecular docking**

The PDB files of YBX1 and ILF3 were obtained from the UniProt database (https://www.uniprot.org/). Preprocessing of these proteins, including residue repair, hydrogen bond optimization, solvent removal, and energy minimization, was performed using AutoDock Tools. Protein-protein docking was then conducted using AutoDock Vina 1.05.36. Visualization and data generation were carried out using PyMOL (educational open-source version) and PDBePISA.

**Bioinformatics analysis**

The expression of different genes in CRC tumor and normal tissues were obtained from the TCGA database (https://portal.gdc.cancer.gov). The GEPIA 2 website (http://gepia2.cancer-pku.cn) was used to analyze gene correlation in CRC. RBPDB (http://rbpdb.ccbr.utoronto.ca/), RBPmap (http://rbpmap.technion.ac.il/), and catRAPID (http://service.tartaglialab.com/) were employed to predict RBPs associated with KSR1 mRNA. catRAPID was also used to predict the interaction region between LINC02167 and YBX1. RNAfold (http://rna.tbi.univie.ac.at) was used to predict the secondary structure of LINC02167. The full-length KSR1 mRNA sequence was retrieved from the NCBI website (NCBI Reference Sequence: NM_014238.2), and the iRNA-m5C ([http://lin-group.cn/server/iRNA-m5C/service.html](https://links.jianshu.com/go?to=https%3A%2F%2Flink.zhihu.com%2F%3Ftarget%3Dhttp%253A%2F%2Flin-group.cn%2Fserver%2FiRNA-m5C%2Fservice.html)) and RNAm5Cfinder (http://rnanut.net/rnam5cfinder) online tools were used to predict potential m5C modification sites along the entire length of the KSR1 mRNA sequence. UCSC (https://genome.ucsc.edu/) and JASPAR (http://jaspar.genereg.net/)were used to predict transcription factors for LINC02167.
