# Supplementary figures and images for: LINC02167 stabilizes KSR1 mRNA in an m5C-dependent manner to regulate the ERK/MAPK signaling pathway and promotes colorectal cancer metastasis
Source: J Exp Clin Cancer Res. 2025 Apr 15;44:121. doi: 10.1186/s13046-025-03368-w (PMC11998267; doi:10.1186/s13046-025-03368-w)

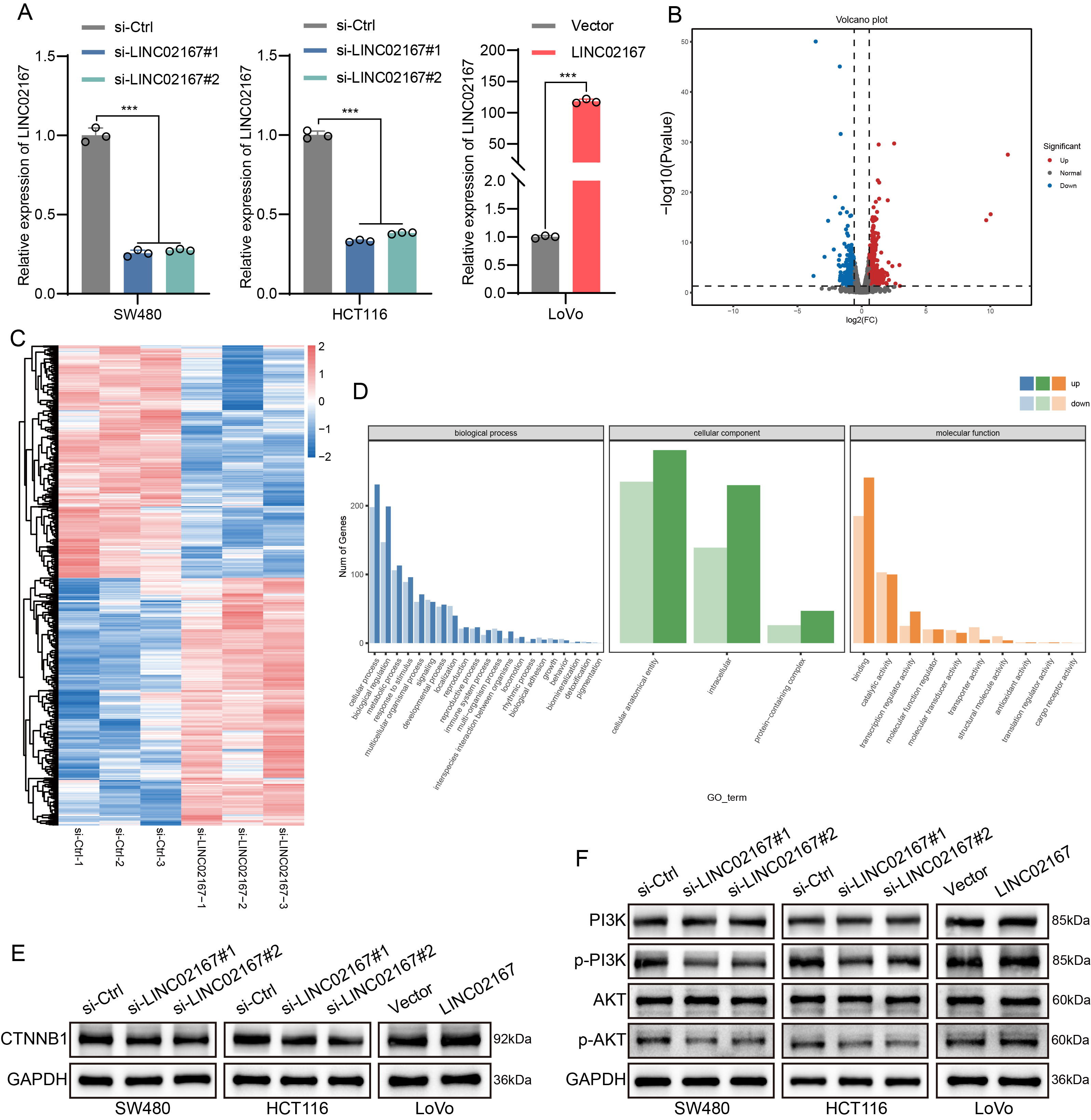

Supplement: Supplementary file 3 — Additional file 3. Fig. S1. (A) Validation of LINC02167 knockdown or overexpression efficiency in CRC cells. (B) Volcano plot showing gene expression differences between control and LINC02167 knockdown groups in CRC cells. (C) Heatmap of differentially expressed genes between control and LINC02167 knockdown groups. (D) Gene Ontology (GO) analysis, including biological process (BP), cellular component (CC), and molecular function (MF), highlighting significantly enriched pathways after LINC02167 knockdown. (E) Western blot analysis showing the effects of LINC02167 knockdown or overexpression on the expression of Wnt signaling pathway-related proteins in CRC cells. (F) Western blot analysis showing the effects of LINC02167 knockdown or overexpression on the expression of PI3K/AKT signaling pathway-related proteins in CRC cells. *P < 0.05, **P < 0.01, ***P < 0.001. [file 13046_2025_3368_MOESM3_ESM.tif]

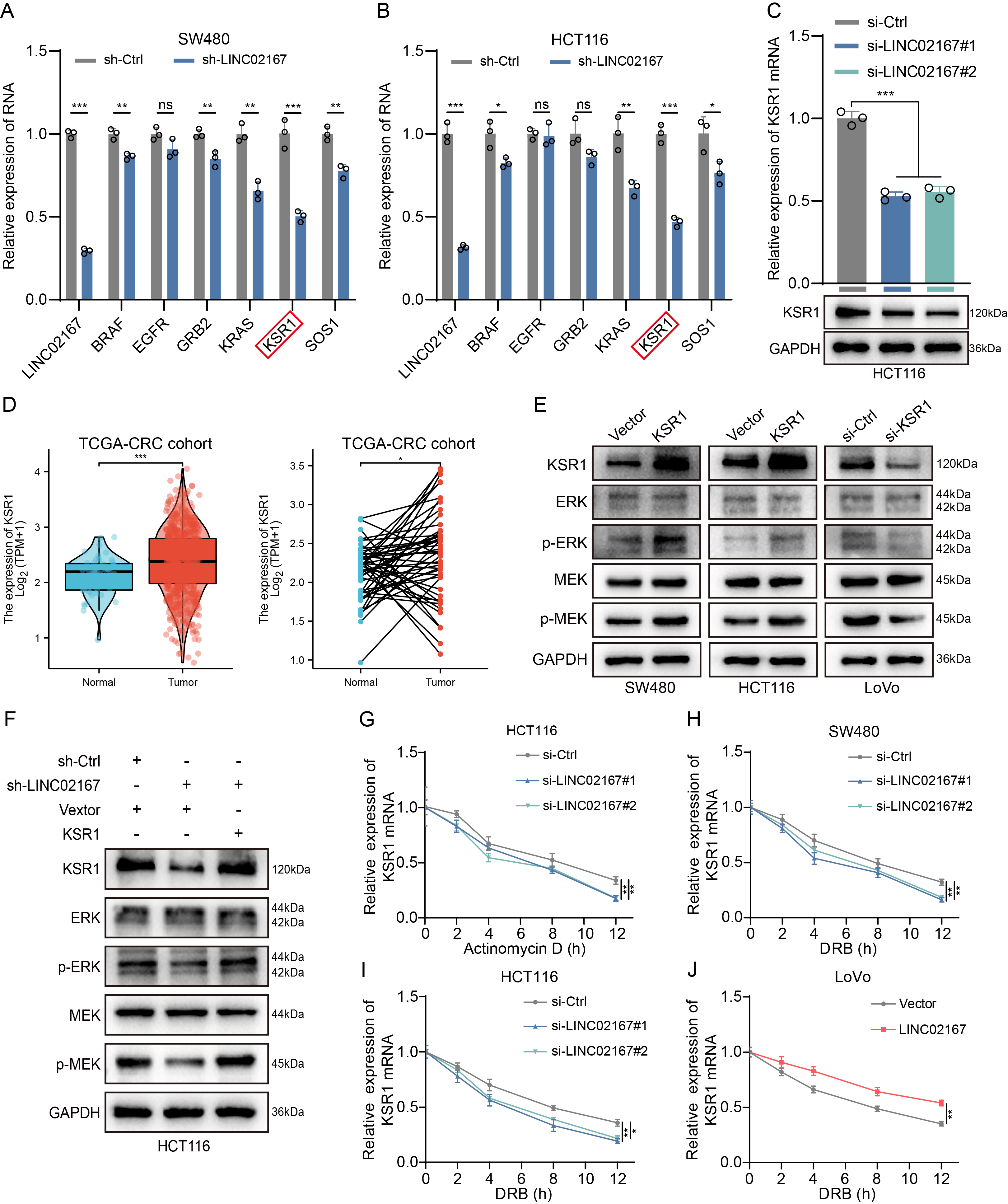

Supplement: Supplementary file 4 — Additional file 4. Fig. S2 (A, B) qRT-PCR analysis of the expression changes in key ERK/MAPK regulatory genes in SW480 and HCT116 cells with stable LINC02167 knockdown. (C) Analysis of KSR1 expression changes after LINC02167 knockdown in HCT116 cells. (D) TCGA database analysis of KSR1 expression in CRC tissues compared to normal tissues. (E) Western blot analysis showing the effect of KSR1 knockdown or overexpression on ERK/MAPK signaling pathway activity. (F) Western blot analysis showing that KSR1 overexpression rescues the reduction in p-ERK and p-MEK levels caused by LINC02167 knockdown in HCT116 cells. (G-I) RNA stability assay showing reduced KSR1 mRNA stability after LINC02167 knockdown in CRC cells. (J) RNA stability analysis showing increased KSR1 mRNA stability following LINC02167 overexpression in LoVo cells. *P < 0.05, **P < 0.01, ***P < 0.001. [file 13046_2025_3368_MOESM4_ESM.tif]

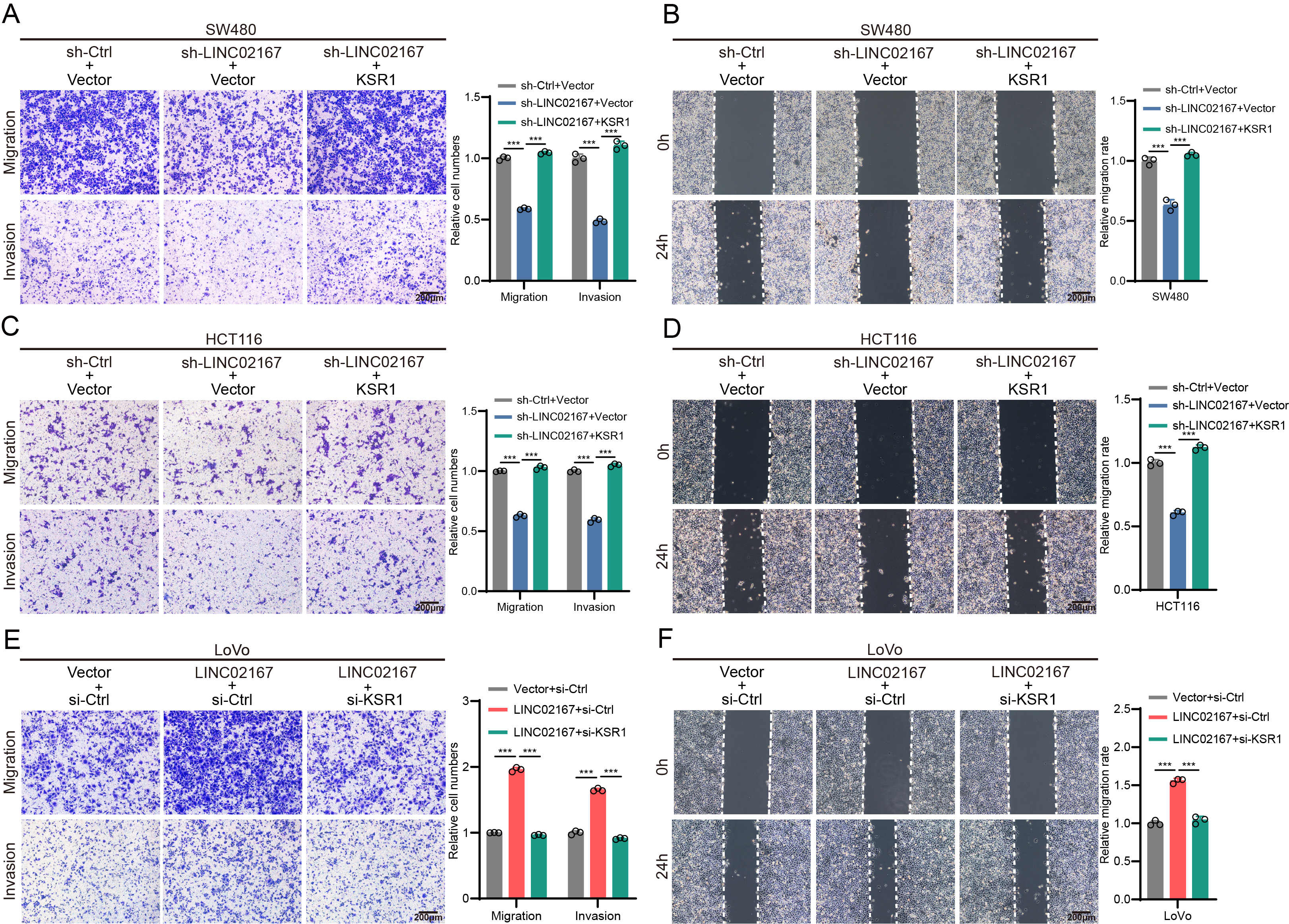

Supplement: Supplementary file 5 — Additional file 5. Fig. S3 (A-D) Transwell assays (A, C) and wound healing assays (B, D) showing that the suppression of CRC cell migration and invasion by LINC02167 knockdown is reversed by KSR1 overexpression. (E, F) Transwell assay (E) and wound healing assay (F) showing that the promotion of CRC cell migration and invasion by LINC02167 overexpression is reversed by KSR1 knockdown. *P < 0.05, **P < 0.01, ***P < 0.001. [file 13046_2025_3368_MOESM5_ESM.tif]

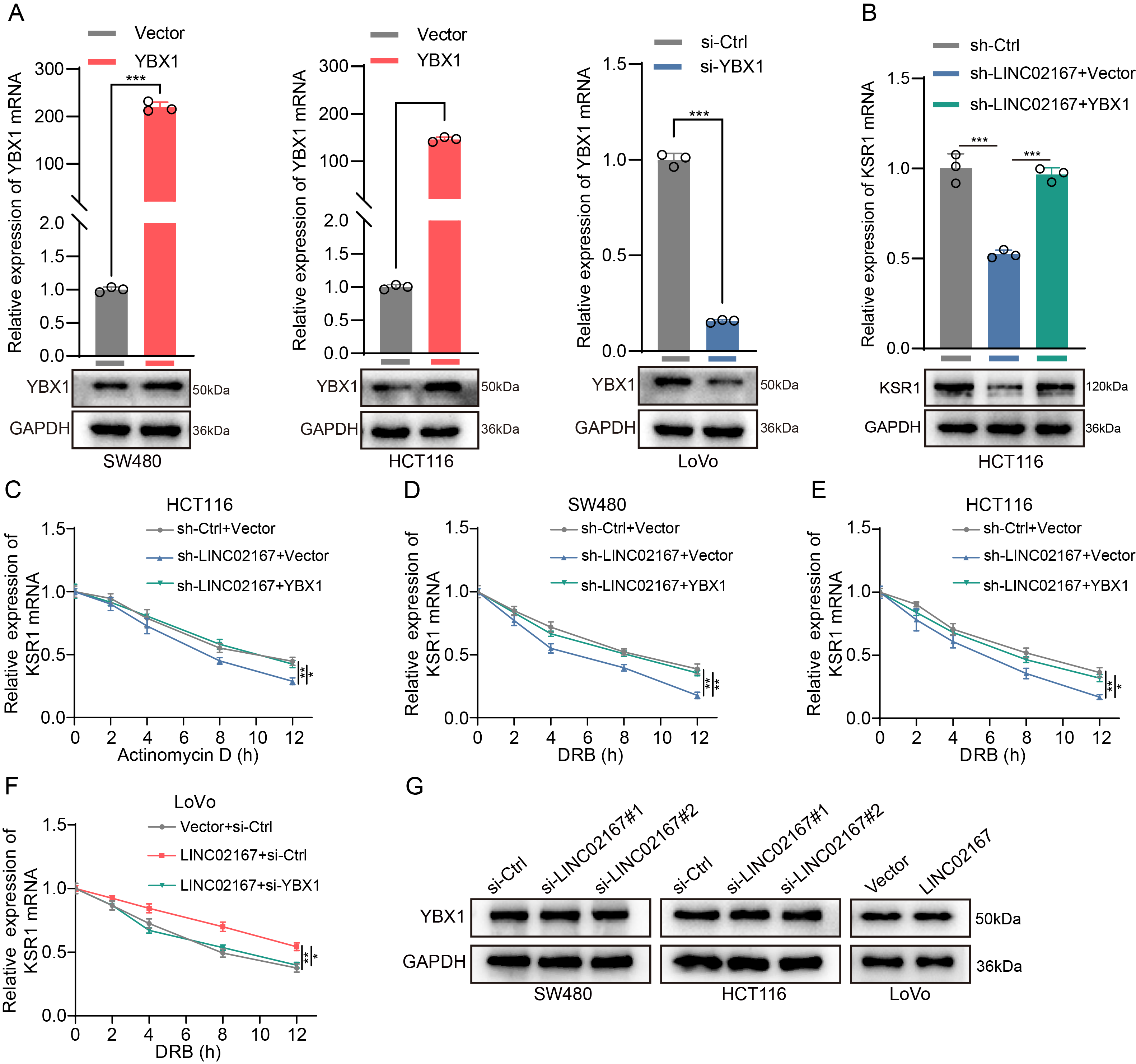

Supplement: Supplementary file 6 — Additional file 6. Fig. S4 (A) Validation of YBX1 knockdown or overexpression efficiency in CRC cells. (B) Analysis of the effect of YBX1 overexpression on KSR1 mRNA and protein expression following LINC02167 knockdown in HCT116 cells. (C-E) RNA stability assay showing that YBX1 overexpression rescues the reduction in KSR1 mRNA stability caused by LINC02167 knockdown in CRC cells. (F) RNA stability analysis showing that YBX1 knockdown reverses the increase in KSR1 mRNA stability caused by LINC02167 overexpression in LoVo cells. (G) Analysis of the effect of LINC02167 knockdown or overexpression on YBX1 protein levels in CRC cells. *P < 0.05, **P < 0.01, ***P < 0.001. [file 13046_2025_3368_MOESM6_ESM.tif]

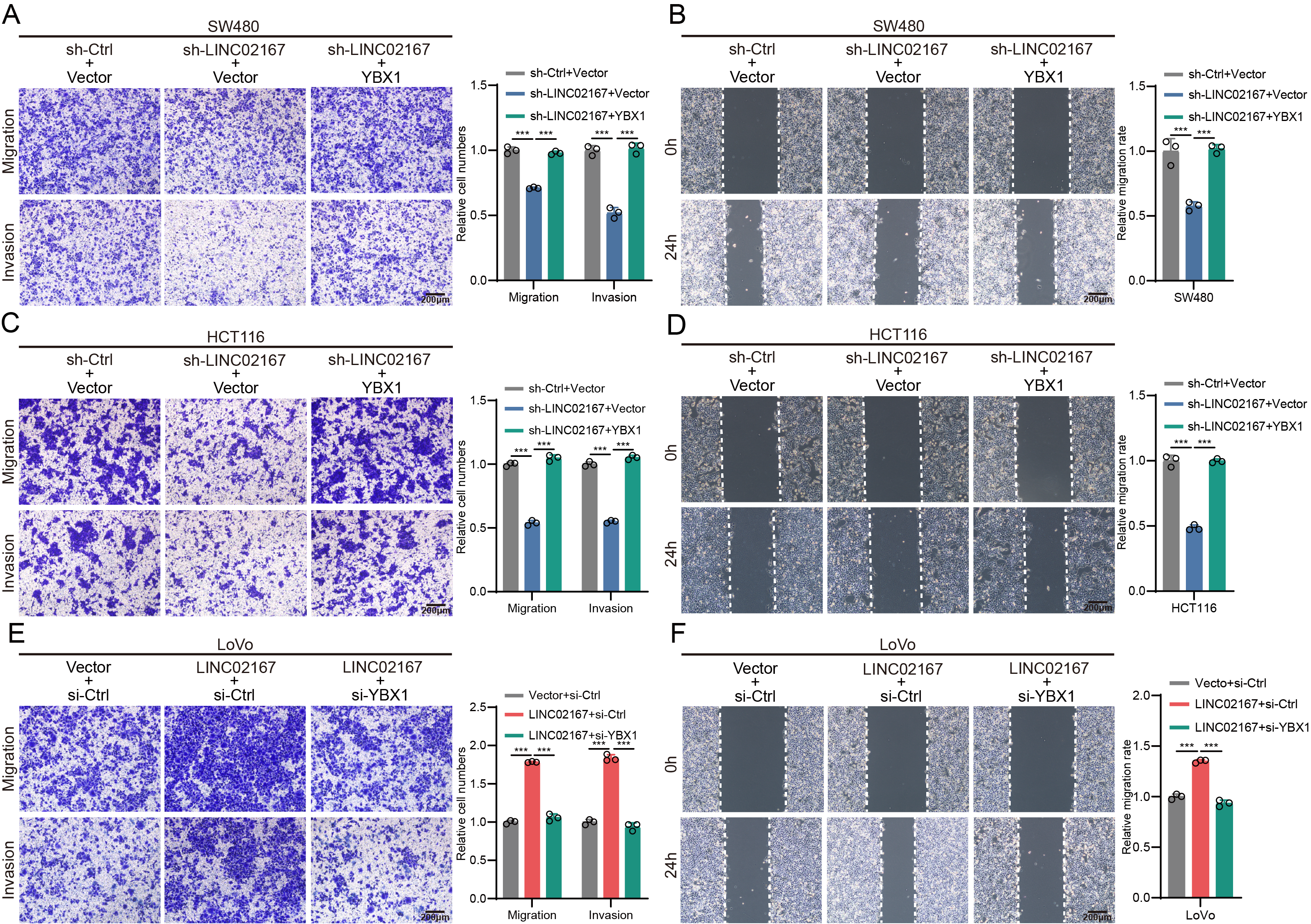

Supplement: Supplementary file 7 — Additional file 7. Fig. S5 (A-D) Transwell assays (A, C) and wound healing assays (B, D) showing that the suppression of CRC cell migration and invasion caused by LINC02167 knockdown is reversed by YBX1 overexpression. (E, F) Transwell assay (E) and wound healing assay (F) showing that the promotion of CRC cell migration and invasion caused by LINC02167 overexpression is reversed by YBX1 knockdown. *P < 0.05, **P < 0.01, ***P < 0.001. [file 13046_2025_3368_MOESM7_ESM.tif]

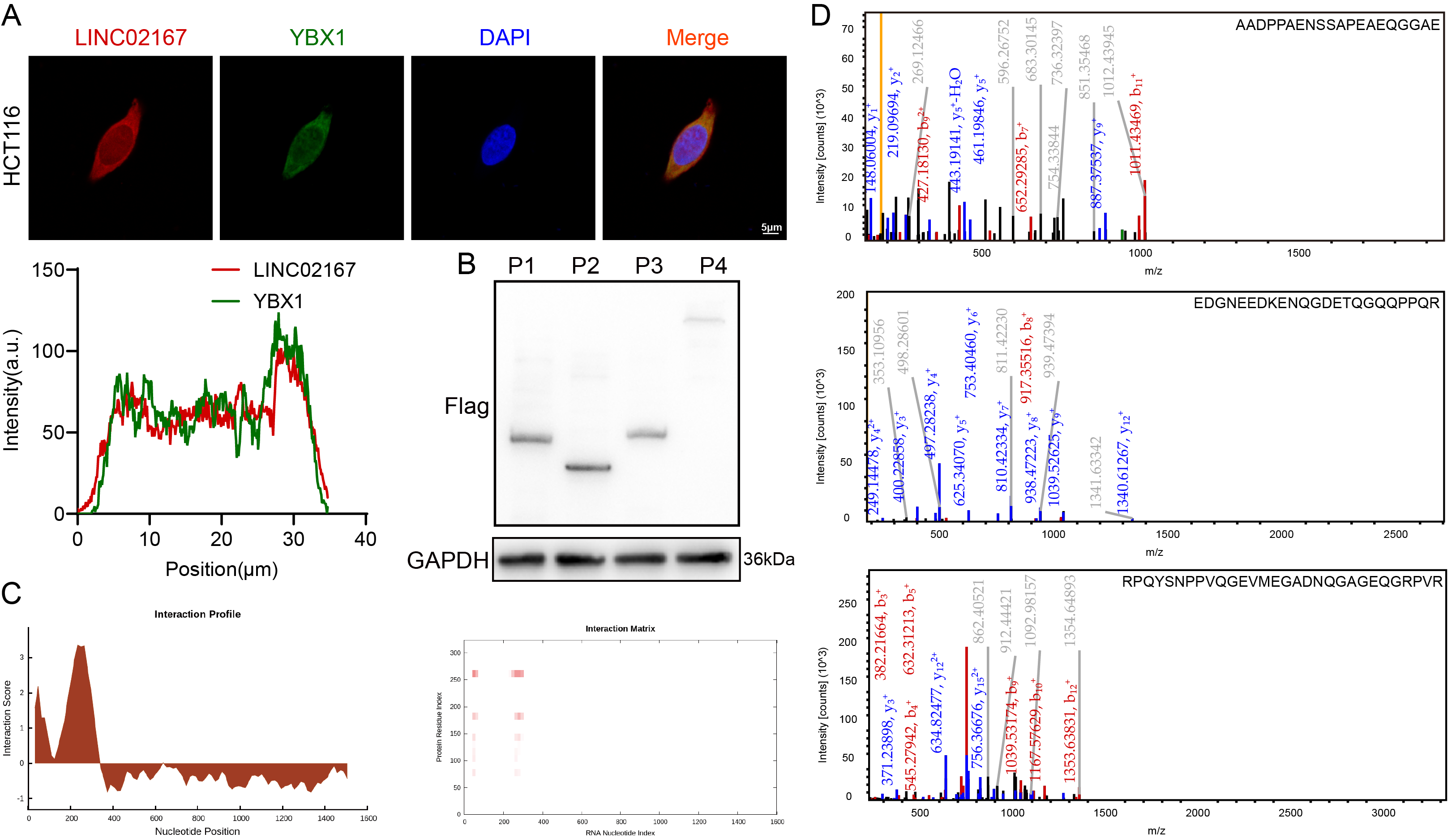

Supplement: Supplementary file 8 — Additional file 8. Fig. S6 (A) Co-localization of LINC02167 (red) and YBX1 (green) in HCT116 cells detected by confocal microscopy. Nuclei were stained with DAPI (blue). (B) Western blot analysis of Flag-tagged YBX1 constructs (P1: 1–129 aa, P2: 130–205 aa, P3: 206–324 aa) transfected into SW480 cells. (C) Predicted interaction regions between LINC02167 and YBX1 based on catRAPID analysis. (D) Representative YBX1 peptides detected by mass spectrometry (MS) as binding to LINC02167 [file 13046_2025_3368_MOESM8_ESM.tif]

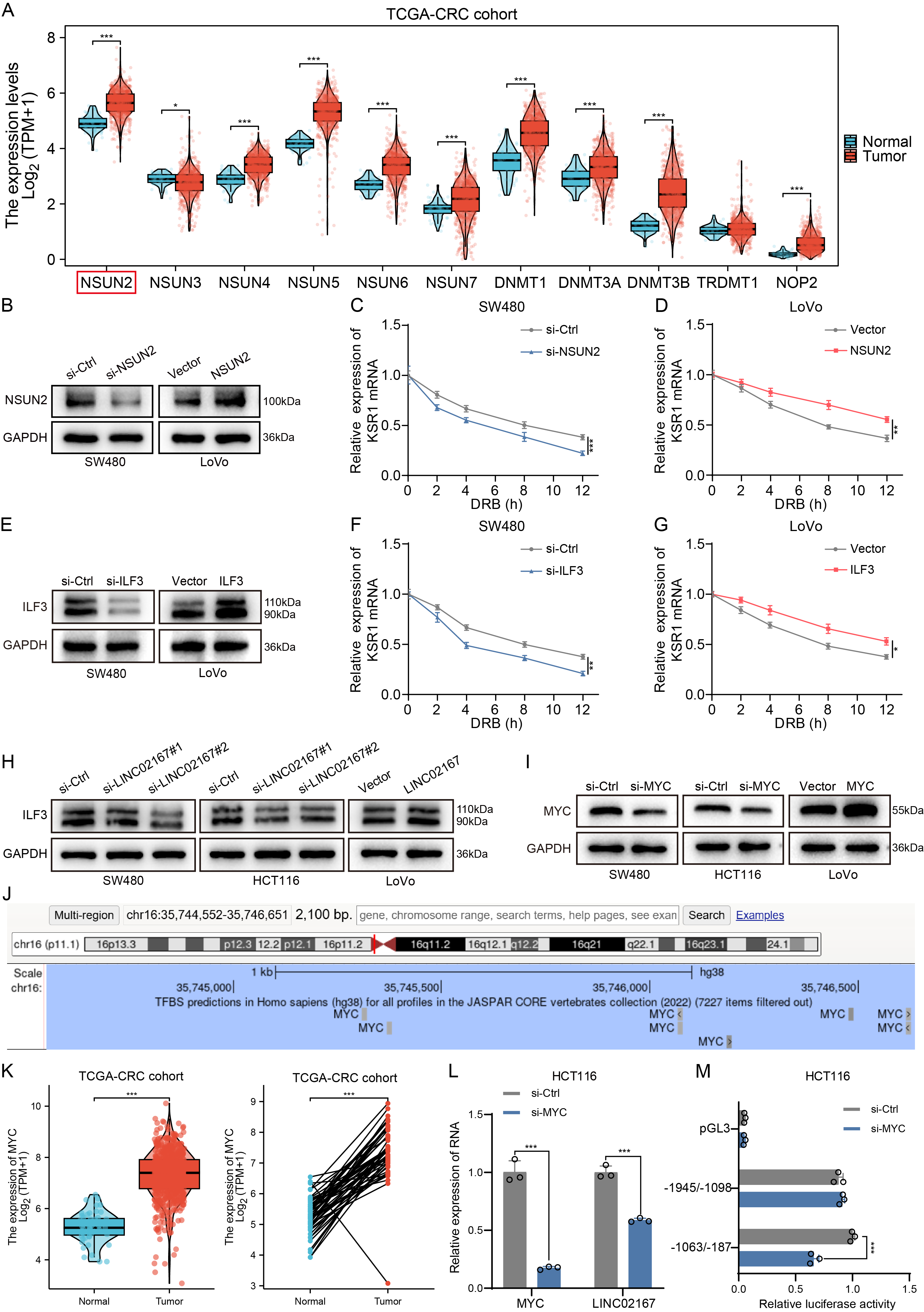

Supplement: Supplementary file 9 — Additional file 9. Fig. S7 (A) Analysis of the expression levels of 11 m5C "Writers" in CRC tissues and normal tissues from the TCGA database. (B) Validation of NSUN2 knockdown or overexpression efficiency in CRC cells. (C, D) RNA stability analysis of KSR1 mRNA stability in CRC cells after NSUN2 knockdown or overexpression. (E) Validation of ILF3 knockdown or overexpression efficiency in CRC cells. (F, G) RNA stability analysis of KSR1 mRNA stability in CRC cells after ILF3 knockdown or overexpression. (H) Analysis of the effect of LINC02167 knockdown or overexpression on ILF3 protein levels in CRC cells. (I) Validation of MYC knockdown or overexpression efficiency in CRC cells. (J) Prediction of LINC02167 transcriptional regulators using the UCSC Genome Browser. (K) Analysis of MYC expression in CRC tissues compared to normal tissues from the TCGA database. (L) Analysis of LINC02167 expression changes after MYC knockdown in HCT116 cells. (M) Luciferase reporter assay showing that knockdown of MYC significantly reduces luciferase activity driven by the LINC02167 promoter (-1063 to -187 fragment) in HCT116 cells. *P < 0.05, **P < 0.01, ***P < 0.001. [file 13046_2025_3368_MOESM9_ESM.tif]
